# Inverted base composition skews and discontinuous mitochondrial genome architecture evolution in the Enoplea (Nematoda)

**DOI:** 10.1186/s12864-022-08607-4

**Published:** 2022-05-18

**Authors:** Hong Zou, Fang-Lin Chen, Wen-Xiang Li, Ming Li, Hong-Peng Lei, Dong Zhang, Ivan Jakovlić, Gui-Tang Wang

**Affiliations:** 1 Key Laboratory of Aquaculture Disease Control, Ministry of Agriculture, and State Key Laboratory of Freshwater Ecology and Biotechnology, Institute of Hydrobiology, Chinese Academy of Sciences, Wuhan, 430072 China; 2 University of Chinese Academy of Sciences, Beijing, 100049 China; 3 State Key Laboratory of Grassland Agro-Ecosystems, and College of Ecology, Lanzhou University, Lanzhou, 730000 China; 4 Bio-Transduction Lab, Wuhan, 430075 China

**Keywords:** Compositional heterogeneity, GC skew, Inversion of the replication order, Gene rearrangement, Mitogenome, Pseudocapillaria tomentosa, Capillariidae, Phylogeny

## Abstract

**Background:**

Within the class Enoplea, the earliest-branching lineages in the phylum Nematoda, the relatively highly conserved ancestral mitochondrial architecture of Trichinellida is in stark contrast to the rapidly evolving architecture of Dorylaimida and Mermithida. To better understand the evolution of mitogenomic architecture in this lineage, we sequenced the mitogenome of a fish parasite *Pseudocapillaria tomentosa* (Trichinellida: Capillariidae) and compared it to all available enoplean mitogenomes.

**Results:**

*P. tomentosa* exhibited highly reduced noncoding regions (the largest was 98 bp), and a unique base composition among the Enoplea. We attributed the latter to the inverted GC skew (0.08) in comparison to the ancestral skew in Trichinellidae (-0.43 to -0.37). Capillariidae, Trichuridae and Longidoridae (Dorylaimida) generally exhibited low negative or low positive skews (-0.1 to 0.1), whereas Mermithidae exhibited fully inverted low skews (0 to 0.05). This is indicative of inversions in the strand replication order or otherwise disrupted replication mechanism in the lineages with reduced/inverted skews. Among the Trichinellida, Trichinellidae and Trichuridae have almost perfectly conserved architecture, whereas Capillariidae exhibit multiple rearrangements of tRNA genes. In contrast, Mermithidae (Mermithida) and Longidoridae (Dorylaimida) exhibit almost no similarity to the ancestral architecture.

**Conclusions:**

Longidoridae exhibited more rearranged mitogenomic architecture than the hypervariable Mermithidae. Similar to the Chromadorea, the evolution of mitochondrial architecture in enoplean nematodes exhibits a strong discontinuity: lineages possessing a mostly conserved architecture over tens of millions of years are interspersed with lineages exhibiting architectural hypervariability. As Longidoridae also have some of the smallest metazoan mitochondrial genomes, they contradict the prediction that compact mitogenomes should be structurally stable. Lineages exhibiting inverted skews appear to represent the intermediate phase between the Trichinellidae (ancestral) and fully derived skews in Chromadorean mitogenomes (GC skews = 0.18 to 0.64). Multiple lines of evidence (CAT-GTR analysis in our study, a majority of previous mitogenomic results, and skew disruption scenarios) support the Dorylaimia split into two sister-clades: Dorylaimida + Mermithida and Trichinellida. However, skew inversions produce strong base composition biases, which can hamper phylogenetic and other evolutionary studies, so enoplean mitogenomes have to be used with utmost care in evolutionary studies.

**Supplementary Information:**

The online version contains supplementary material available at 10.1186/s12864-022-08607-4.

## Background

The class Enoplea comprises the earliest-branching lineages in the phylum Nematoda. It is divided into two subclasses: Dorylaimia (or Clade I) and Enoplia (Clade II) [1]. Dorylaimia exhibit a diverse life history range: the vertebrate-parasitic order Trichinellida, the insect-parasitic Mermithida, the plant-parasitic Dorylaimida, and the free-living Mononchida [1]. Previously sequenced enoplean mitochondrial genomes (mitogenomes) suggest that the evolution of mitochondrial architecture in this clade exhibits a rather stark discontinuity: Trichinellidae and Trichuridae families of the order Trichinellida appear to possess a perfectly conserved architecture [2–5], Dorylaimida (Longidoridae) exhibit a fast-evolving mitogenomic architecture [5, 6], and Mermithidae (Mermithida) exhibit a rampant gene order rearrangement rate, unmatched within the Bilateria [7, 8]. This pattern fits our previous observation that mitogenome architecture evolution is discontinuous (in nematodes) and that long evolutionary periods of stasis are interspersed with lineages that exhibit exponentially accelerated mitochondrial evolution rates [9]. However, several key enoplean lineages remain unrepresented in terms of available mitogenomes, e.g. the entire Enoplia and Mononchida lineages, which limits our understanding of the evolution of mitochondrial architecture within this nematode lineage. 

The Nematoda has been recognised as a problematic lineage in bilaterian/ecdysozoan mitochondrial phylogenomics [10, 11]. The phylogeny of Enoplea also remains unresolved, largely because genomic data (both mitogenomic and nuclear) remain unavailable for many key enoplean lineages, but also because previous mitogenomic studies of Dorylaimia produced inconsistent results [6, 12, 13]. Base composition biases may cause strong artefacts in phylogenetic analyses [14–18], and mitochondrial genomes often exhibit strong compositional biases as a consequence of directional mutational pressures predominantly associated with mitochondrial replication [19, 20]. During the replication, the parental H-strand is left in a mutagenic single-stranded state for almost two hours [21], which may cause spontaneous hydrolytic deamination of A and C (into G and T respectively). Over multiple generations, this results in the accumulation of T and G on the H-strand, and A and C on the L-strand [19]. In some cases, mitochondrial architecture rearrangements may result in the inversion of the origin of replication, which then also causes an inversion of the direction of mutational pressures, i.e., now T and G accumulate on the L-strand, and A and C on the H-strand [22]. Such skew inversions can affect the branch length, mutational saturation, codon usage, protein properties, and cause reverse mutations, so they can interfere with a broad range of evolutionary studies [16].

*Pseudocapillaria tomentosa* is a widespread intestinal nematode parasite found in the intestines of a broad range of fish hosts in the Northern Hemisphere [23, 24]. Until relatively recently, this parasite was very poorly studied [23], but this species often parasitizes zebrafish, so along with the relatively rapid increase in the popularity of zebrafish as a model animal for molecular biology, this nematode concomitantly received somewhat increased scientific attention in recent years [25–27]. Despite this, molecular data for *P. tomentosa* remain almost completely unavailable. The only currently (July 2021) available GenBank entry is a partial *18S* rRNA gene sequence (KU987805) [23]. Furthermore, at the onset of this study, there were no mitogenomes available for the entire family Capillariidae (Trichinellida). In the meantime, two sequences were submitted to the GenBank: the mitogenome of a fish parasite *Eucoleus annulatus*, collected in the USA [12], and the mitogenome of a cat parasite *Capillaria* sp. (Australia) (MH665363; unpublished).

For this study, we sequenced the complete mitogenome of *Pseudocapillaria tomentosa* (Dujardin, 1843) Moravec, 1987 (Dorylaimia: Trichinellida: Capillariidae) to generate molecular data necessary for the identification, population studies, and phylogeny of this species and family, as well as to test two working hypotheses: 1. mitogenomic architecture evolution is discontinuous in the Enoplea, and 2. base composition biases are hampering mitochondrial phylogenomic reconstruction in the Enoplea. Along with *P. tomentosa*, we used all mitogenomic data available for the Enoplea to conduct detailed comparative mitogenomic analyses and test the two hypotheses.

## Results and Discussion

### General mitogenomic features of *P. tomentosa* and Capillariidae

**Fig. 1:**
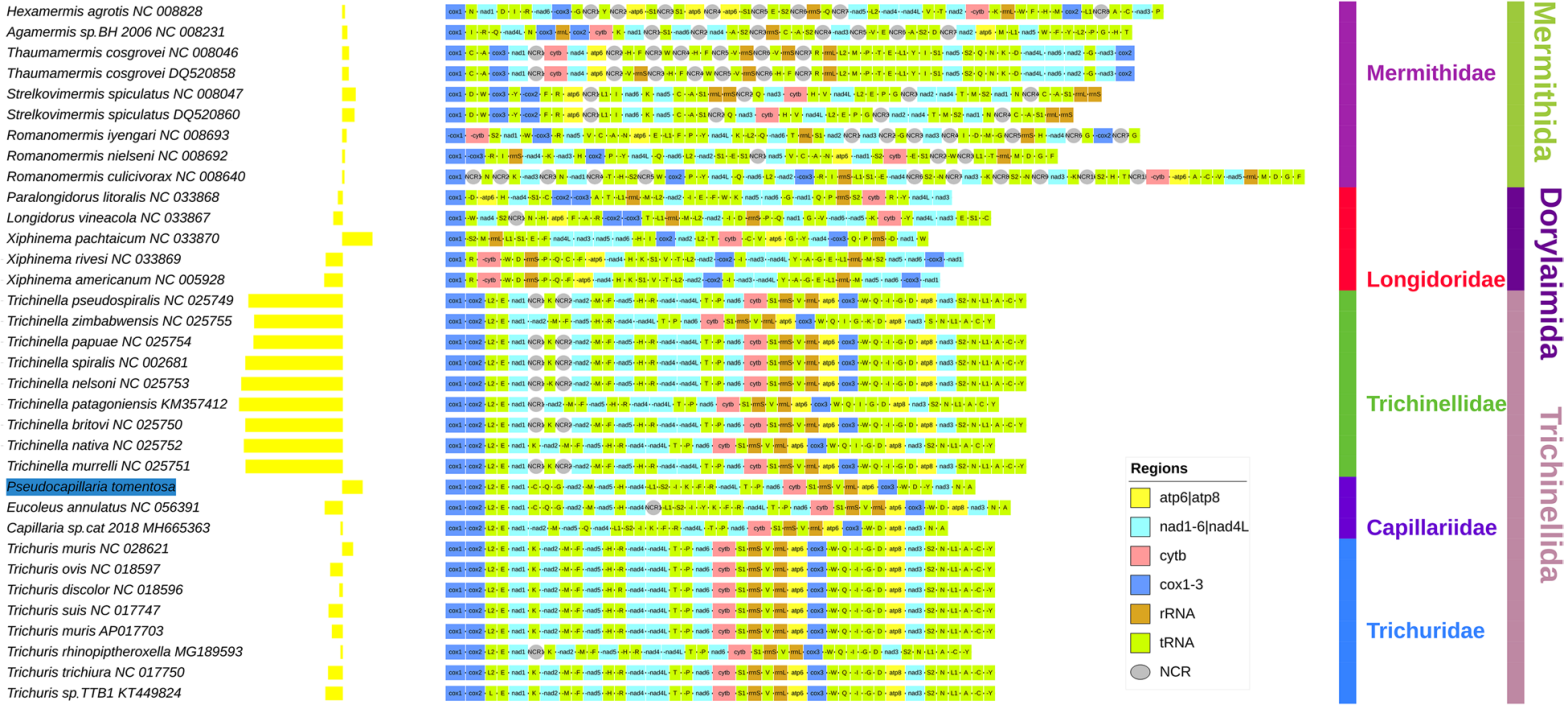
Mitochondrial architecture and skews of enoplean mitogenomes. GC skews on the plus strand are shown as yellow bars, where negative values are to the left from the axis and positive to the right. The legend for the mitochondrial architecture is shown in the figure, with only NCRs larger than 200 bp shown. Species names are given with GenBank accession numbers. The family and order-level taxonomic identity is shown to the right. *Pseudocapillaria tomentosa* is highlighted in blue

The complete mitogenome of *P. tomentosa* contained all 37 standard mitochondrial genes, which were encoded on both strands (Fig. 1; for additional results and discussion see Additional file 1). This is common for the Enoplea, whereas in Chromadorea genes are encoded on a single strand [28]. The strand distribution of protein-coding genes (PCGs) was conserved among the Trichinellida, with *nad2, nad5, nad4* and *nad4L* consistently encoded on the minus strand, but variable among the Dorylaimida and Mermithida (Fig. 1). In the mitogenome of *P. tomentosa*, a majority of genes kept the transcription sense: a stretch of 16 genes on the minus strand was punctuated only by *trnK* and *trnT*. *trnW* was the only gene encoded on the minus strand that was isolated from this main stretch. Start codons (ATG, ATT, ATA) and stop codons (TAA and TAG) were standard. Gene overlaps were few (4) and small (1 to 2 bp). The distribution of tRNA genes was conserved among most Trichinellida, apart from the Capillariidae. In this family, the architecture was conserved between *P. tomentosa* and *E. annulatus* [12], whereas *Capillaria* sp. appears to exhibit a slightly rearranged architecture, but these may be sequencing and annotation artefacts as the mitogenome is almost certainly incomplete (Additional file 1). In comparison to *E. annulatus*, *P. tomentosa* exhibited an identical gene order and strand distribution, but only partially conserved codon usage, low gene sequence similarity (average identity = 64.65%), partially conserved distribution of intergenic regions, and almost no similarity in the distribution of gene overlaps (Table 1).

**Table 1:** The comparison of mitochondrial architectures of *Eucoleus annulatus* (left) and *Pseudocapillaria tomentosa* (right)

Gene	Position		Size	IGN	Codon		Strand	Identity
	From	To			Start	Stop		
*cox1*	1/1	1548/1548	1548/1548		ATG/ATG	TAA/TAA	H/H	76.42
*cox2*	1570/1554	2253/2234	684/681	21/5	ATG/ATG	TAA/TAA	H/H	68.13
*trnL2*	2262/2252	2325/2319	64/68	8/17			H/H	70.59
*trnE*	2335/2319	2391/2374	57/56	9/-1			H/H	73.68
*nad1*	2438/2400	3337/3293	900/894	46/25	ATG/ATT	TAA/TAA	H/H	68.56
*trnC*	3383/3392	3440/3441	58/50	45/98			L/L	58.62
*trnQ*	3423/3446	3498/3500	76/55	-18/4			L/L	63.16
*trnG*	3546/3515	3620/3568	75/54	47/14			L/L	49.33
*nad2*	3676/3619	4578/4518	903/900	55/50	ATT/ATA	TAA/TAG	L/L	65.15
*trnM*	4579/4519	4640/4580	62/62				L/L	85.48
*nad5*	4655/4579	6214/6120	1560/1542	14/-2	ATA/ATA	TAA/TAG	L/L	62.18
*trnH*	6215/6121	6270/6177	56/57				L/L	64.41
*nad4*	6275/6228	7228/7487	954/1260	4/50	ATT/ATT	TAA/TAA	L/L	48.57
*NCR*	7229/–	7534/–	306/–				NA	
*trnL1*	7535/7489	7599/7557	65/69	–/1			L/L	71.43
*trnS2*	7600/7576	7654/7629	55/54	–/18			L/L	30.99
*trnI*	7654/7703	7722/7767	69/65	-1/73			L/L	71.01
*trnY*	7728/7766	7786/7824	59/59	5/-2			L/L	62.71
*trnK*	7834/7849	7904/7911	71/63	47/24			H/H	68.06
*trnF*	7893/7927	7967/7982	75/56	-12/15			L/L	61.33
*trnR*	7964/7988	8026/8054	63/67	-4/5			L/L	64.71
*nad4L*	8032/8058	8268/8306	237/249	5/3	ATA/ATT	TAA/TAA	L/L	66.67
*trnT*	8282/8308	8338/8361	57/54	13/1			H/H	77.19
*trnP*	8328/8374	8399/8427	72/54	-11/12			L/L	70.83
*nad6*	8400/8429	8860/8884	461/456	–/1	TTG/ATT	TA/TAG	H/H	66.67
*cytb*	8861/8898	9973/10010	1113/1113	–/13	ATG/ATG	TAA/TAA	H/H	73.23
*trnS1*	9974/10010	10,038/10063	65/54	–/-1			H/H	55.07
*rrnS*	10,039/10064	10,852/10739	814/676				H/H	61.88
*trnV*	10,853/10740	10,909/10793	57/54				H/H	58.62
*rrnL*	10,910/10794	11,751/11734	842/941				H/H	60.78
*atp6*	11,752/11735	12,540/12532	789/798		ATA/ATT	TAA/TAA	H/H	66.79
*cox3*	12,541/12538	13,317/13311	777/774	–/5	ATG/ATG	TAA/TAA	H/H	66.41
*trnW*	13,336/13315	13,398/13376	63/62	18/3			L/L	84.13
*trnD*	13,402/13378	13,456/13432	55/55	3/1			H/H	72.73
*atp8*	13,463/13433	13,603/13579	141/147	6/–	ATG/ATC	TAA/TAA	H/H	51.35
*nad3*	13,631/13588	13,960/13929	330/342	27/8	ATA/ATA	TAA/TAA	H/H	66.08
*trnN*	13,985/13934	14,044/13993	60/60	24/4			H/H	49.32
*trnA*	14,044/14009	14,099/14062	56/54	-1/15			H/H	59.68

IGN column shows the sizes of intergenic regions (positive values) or gene overlaps (negative values) in base pairs

### Gene order

We numerically assessed our observation that the evolution of mitochondrial architecture in enoplean nematodes appears to be discontinuous using the common intervals gene order similarity measure (where the value 0 indicates a complete absence of shared common intervals) [29]. Almost all Trichinellidae and Trichuridae exhibited an identical gene order (Table 2). The only exceptions were *Trichinella zimbabwensis*, which exhibited a translocation of the *trnK* gene, and *Trichinella patagoniensis*, which exhibited a loss of the *trnK* gene. Both of these might be sequencing or annotation artefacts. Trichinellidae exhibit a great similarity to the ancestral arthropod mitochondrial architecture (gene arrangement), which is also similar to the ancestral ecdysozoan mitogenome architecture [2, 30–32]. Trichinellidae are therefore considered to possess the most ancestral architecture among all nematodes, whereas all other lineages exhibit highly derived architectures, so we will treat this gene order as the ancestral for all Nematoda. In comparison to the ancestral gene order, Capillariidae exhibited a large number of rearrangements (Fig. 1, Additional file 1), but within the enoplean dataset, they exhibited an intermediate gene rearrangement rate (168—178). Strongly accelerated evolutionary rates were observed in Mermithidae, which exhibited very little similarity to the ancestral gene order (2—12), and Longidoridae, which exhibited almost no similarity to the ancestral gene order (0 – 2). This partially corroborates the results of visual assessment, but surprisingly indicates that Longidoridae, and not Mermithidae, possess the most highly rearranged gene orders. The explanation is that rearrangements in Mermithidae are concentrated in specific, hypervariable regions [7], whereas some segments remain conserved. This is not the case in Longidoridae, which do not exhibit any conserved segments. Intriguingly, Longidoridae (*Xiphinema*) species have some of the smallest metazoan mitochondrial genomes sequenced so far [5, 6], which contradicts the proposition that compact mitogenomes are structurally stable, i.e. that shorter mitogenomes exhibit fewer gene order rearrangements relative to the ancestral state [33]. Indeed, it is expected that rearrangements should produce an increase in the number of noncoding regions, regardless of whether the underlying mechanism is tandem duplication/random loss [34] of recombination [35]. In agreement with this, Mermithidae, where the mechanism producing the mitogenomic architecture variability is believed to be recombination, exhibit multiple large (> 100 bp) NCR and some of the largest mitogenomes among the Bilateria [7, 8]. Therefore, this is an intriguing discrepancy, which was not discussed by previous studies (to our knowledge) [5, 6]. There are two possible mechanisms to explain it: 1) Longidoridae is undergoing a strong purifying selection directed towards the reduction of mitogenome size, 2) The mechanism of rearrangement is unique in this lineage, and it does not produce multiple NCRs. It should be noted that Longidoridae also exhibit a high level of within-family rearrangement rates (Table 2), which implies that the first scenario would require very strong purifying selection levels. A possible explanation could be found in the ‘race for replication’ hypothesis, which proposes that high metabolic demands impose stronger purifying selection constraints for small genome size [36], but it is unclear why Longidoridae would have higher metabolic demands than other enoplean nematodes. Further studies, and more mitogenomes, are needed to elucidate the rearrangement mechanisms in this family and the evolutionary pressures producing the small mitogenome size.

**Table 2:** Gene order distances in the class Enoplea

	T + T	Tz	Tp	Csp	Pt	Xa	Xp	Lv	Pl	Tc1	Tc2	Ss1	Ss2	Asp	Rc	Rn	Ri	Ha
Trichinellidae and Trichuridae																		
Ancestral GO		640	1254	178	168	2	0	2	0	8	8	6	6	2	8	4	12	8
* Trichinella zimbabwensis*	640		1184	214	174	2	0	0	0	10	10	6	6	0	4	2	8	8
* Trichinella patagoniensis*	1254	1184		206	168	2	0	0	0	8	8	6	6	0	8	4	12	8
Capillariidae																		
* Capillaria* sp	178	214	206		872	4	2	0	2	10	10	10	10	2	2	2	6	4
* Pseudocapillaria tomentosa*	168	174	168	872		6	2	0	2	10	10	12	12	0	2	2	8	0
Longidoridae																		
* Xiphinema americanum*	2	2	2	4	6		50	36	28	6	10	4	2	0	12	8	8	6
* Xiphinema pachtaicum*	0	0	0	2	2	50		30	20	2	0	10	6	0	24	10	6	8
* Longidorus vineacola*	2	0	0	0	0	36	30		110	6	10	4	4	2	14	14	4	6
* Paralongidorus litoralis*	0	0	0	2	2	28	20	110		8	12	6	6	4	6	14	6	4
Mermithidae																		
* Thaumamermis cosgrovei 1*	8	10	8	10	10	6	2	6	8		942	4	4	2	4	2	12	8
* Thaumamermis cosgrovei 2*	8	10	8	10	10	10	0	10	12	942		4	4	2	4	2	10	8
* Strelkovimermis spiculatus 1*	6	6	6	10	12	4	10	4	6	4	4		684	2	2	4	8	8
* Strelkovimermis spiculatus 2*	6	6	6	10	12	2	6	4	6	4	4	684		2	2	4	6	6
* Agamermis* sp	2	0	0	2	0	0	0	2	4	2	2	2	2		6	4	0	10
* Romanomermis culicivorax*	8	4	8	2	2	12	24	14	6	4	4	2	2	6		130	28	6
* Romanomermis nielseni*	4	2	4	2	2	8	10	14	14	2	2	4	4	4	130		58	4
* Romanomermis iyengari*	12	8	12	6	8	8	6	4	6	12	10	8	6	0	28	58		4
* Hexamermis agrotis*	8	8	8	4	0	6	8	6	4	8	8	8	6	10	6	4	4	

The distances were inferred using the Common Intervals measure in CREx, where a value of 0 indicates no similarity, whereas numbers over 1,000 indicate almost perfectly conserved GO. Column headers mirror row headers, but names are acronymic. Only unique architectures are shown, and species are clustered in the corresponding families. Ancestral GO row comprises all Trichinellidae and Trichuridae mitogenomes, apart from the two *Trichinella* species below. *Pseudocapillaria tomentosa* is equal to *Eucoleus annulatus*, and *Xiphinema rivesi* is equal to *Xiphinema americanum*

Until relatively recently the mitogenomic architectural variability was believed to be concentrated largely within the enoplean family Mermithidae, whereas Chromadorea, which contains the majority of Nematoda species, was believed to possess a relatively stable mitogenomic architecture [7, 13, 28, 37–39]. In our previous study, we have shown that some Chromadorean lineages also exhibit exponentially accelerated rates of mitogenomic architecture evolution, which prompted us to propose that the mitogenomic architecture evolution is discontinuous in nematodes [9]. Our analyses conducted for this study indicate that the evolution of mitogenomic architecture is nonlinear in the class Enoplea as well.

### A strongly reduced size of noncoding regions

The newly sequenced mitogenome exhibited a remarkably small largest noncoding region of 98 base pairs (bp) (Fig. 1; Table 1). Normally, metazoan mitogenomes possess a large noncoding region, commonly spanning > 500 bp to a few thousand bp, which usually comprises the control region (CR). For example, in the closely related Trichinellidae, the putative CR spans 700 to 1600 bp, and it is duplicated in most species [2, 3] (Fig. 1; see Additional file 1 for discussion of putative sequencing artefacts). Mermithidae exhibited on average about eight large (> 100 bp) NCRs, but some *Romanomermis* species had more than ten of them [40]. This is attributed to the disrupted, rapidly evolving, mitogenomic architecture in this family, comprising multiple duplicated genes and some of the largest mitogenomes among the nematodes [7]. Surprisingly, the longest NCR (putative CR) in the mitogenome of *T. cosgrovei* is also reduced in size (401 bp) in comparison to standard metazoan mitogenomes [7]. Some Longidoridae (*Xiphinema*) species are known to have some of the smallest metazoan mitochondrial genomes [5, 6], as a result of shortened genes, short noncoding regions, and gene overlaps [5]. However, the mitogenome (14,118 bp) and protein-coding genes (PCGs) of *P. tomentosa* were not reduced in size in comparison to other Trichinellida species, and the overlaps were few and small. Therefore, only the noncoding regions are strongly reduced in size in *P. tomentosa*. In Capillariidae and Trichuridae, only *E. annulatus* and *Trichuris rhinopiptheroxella* had an NCR larger than 200 bp (306 and 208 bp, respectively; see Additional file 1 for additional discussion). However, despite perfectly matching gene arrangements between *P. tomentosa* and *E. annulatus*, the former mitogenome exhibited a negligible intergenic region of 1 bp in the location corresponding to the location of the putative CR of *E. annulatus* (306 bp NCR between *nad4* and *trnL1*) (Fig. 1; Additional file 1). This indicates that the putative CR is rapidly evolving in this family, which makes its identification very difficult. *Trichuris* species largely exhibit two “major” NCRs, both of which are also smaller than 200 bp: the longer one tends to be in the range of 120–170 bp, whereas the short one tends to be 90—120 bp [4, 41]. In conclusion, NCRs are normal-sized in Trichinellidae, slightly reduced in size but multiplied due to rearranged architecture in Mermithidae, and strongly reduced in the Longidoridae, Capilariidae and Trichuridae.

### Base composition and skews

High AT content is common in the mitogenomes of nematodes [28], but *P. tomentosa* exhibited the highest AT bias among all available Trichinellida (79.3%). Among the Dorylaimia, only three Mermithidae species had marginally higher AT content (*Agamermis* and *Romanomermis* sp.; Table 3). As the content of A and G was average, the exceptional AT content is attributable to the very high T content (41.3%), with only three Mermithidae species exhibiting higher values, and to exceptionally low C content, which was the lowest in the entire dataset: 9.5%. This made us suspect that the unique base composition of this species may be associated with mutational pressures associated with the mitogenomic replication mechanism [16, 19, 22]. To assess this, we inspected their base composition skews (GC) [42]. All Trichinellidae mitogenomes exhibited high negative GC skews (skews were calculated for the entire plus strand, and we refer to skew magnitude in absolute terms, i.e. distance from zero, throughout the manuscript) from -0.431 to -0.368. The remaining Trichinellida lineages, comprising Trichuridae and Capillariidae, exhibited low negative skews (-0.073 to -0.007), with *Trichuris muris* (0.043) and *P. tomentosa* (0.083) even exhibiting fully inverted (positive) skews. Dorylaimida (Longidoridae) exhibited a very similar pattern: low negative skews (-0.075 to -0.018), with *Xiphinema pachtaicum* exhibiting a fully inverted skew (0.124). Finally, all available Mermithida (Mermithidae) species exhibited fully inverted (positive) but low skews in the range between 0.007 and 0.054. After *X. pachtaicum*, *P. tomentosa* exhibited the second-highest positive skew in the dataset. The patterns produced by cumulative skew plots were inconsistent, even among some closely related taxa, such as *P. tomentosa* and *Capillaria* sp. (Additional file 1). Apart from Trichinellidae, most species (including *P. tomentosa* and *E. annulatus*) exhibited rather noisy patterns, with skew plots switching between positive and negative values.

**Table 3:** Comparative mitochondrial architecture in the Dorylaimia subclass

Organism	ID	Length	A	T	C	G	A + T	G + C	GC	GC_NCR
Dorylaimida: Longidoridae										
* Xiphinema pachtaicum*	NC_033870	12,489	29.4	39.1	13.7	17.6	68.5	31.3	0.124	0.115
* Paralongidorus litoralis*	NC_033868	12,763	32.4	31.5	18.4	17.7	63.9	36.1	-0.018	0.007
* Xiphinema rivesi*	NC_033869	12,624	37.4	31.5	16.6	14.5	68.9	31.1	-0.069	0
* Xiphinema americanum*	NC_005928	12,626	36.6	29.9	18	15.5	66.5	33.5	-0.075	-0.127
* Longidorus vineacola*	NC_033867	13,519	31.7	32	18.8	17.5	63.7	36.3	-0.037	-0.128
Mermithida: Mermithidae										
* Strelkovimermis spiculatus*	NC_008047	18,030	36	42.5	10.1	11.3	78.5	21.4	0.054	0.047
* Strelkovimermis spiculatus*	DQ520860	17,118	35.9	42.7	10.2	11.2	78.6	21.4	0.049	0.047
* Romanomermis iyengari*	NC_008693	18,919	39.3	40.2	10.1	10.4	79.5	20.5	0.016	0.03
* Thaumamermis cosgrovei*	NC_008046	20,013	32.1	39.3	13.9	14.7	71.4	28.6	0.026	0.011
* Thaumamermis cosgrovei*	DQ520858	21,506	31.9	39.4	14	14.7	71.3	28.7	0.025	0.008
* Hexamermis agrotis*	NC_008828	24,606	41	37.4	10.7	10.9	78.4	21.6	0.009	0.002
* Romanomermis nielseni*	NC_008692	15,546	38.8	40.3	10.3	10.5	79.1	20.8	0.009	0.001
* Romanomermis culicivorax*	NC_008640	26,194	41.3	38	10.3	10.4	79.3	20.7	0.007	-0.014
* Agamermis sp.*	NC_008231	16,561	36.8	43.7	9.6	9.9	80.5	19.5	0.019	-0.02
Trichinellida: Capillariidae										
* Pseudocapillaria tomentosa*		14,062	38	41.3	9.5	11.2	79.3	20.7	0.083	0.032
* Eucoleus annulatus*	NC_056391	14,118	38.3	38.3	12.6	10.9	76.6	23.5	-0.073	-0.288
* Capillaria sp. cat-2018*	MH665363	13,624	37	39	12.1	11.9	76	24	-0.007	-0.391
Trichinellida: Trichinellidae										
* Trichinella murrelli*	NC_025751	16,592	40.7	26.8	22.9	9.7	67.5	32.6	-0.404	-0.37
* Trichinella spiralis*	NC_002681	16,706	40.5	26.5	23	9.7	67	32.7	-0.405	-0.371
* Trichinella britovi*	NC_025750	16,421	40.6	26.6	23.1	9.8	67.2	32.9	-0.405	-0.395
* Trichinella papuae*	NC_025754	17,326	40.2	26.6	22.7	10.4	66.8	33.1	-0.371	-0.412
* Trichinella pseudospiralis*	NC_025749	17,667	40.9	26.6	22.6	9.9	67.5	32.5	-0.392	-0.425
* Trichinella nelsoni*	NC_025753	15,278	40.6	25.5	24.1	9.8	66.1	33.9	-0.422	-0.46
* Trichinella patagoniensis*	KM357412	15,179	40.1	24.9	24.4	9.7	65	34.1	-0.431	-0.485
* Trichinella zimbabwensis*	NC_025755	14,244	39.6	25.4	24	11.1	65	35.1	-0.368	-0.502
* Trichinella nativa*	NC_025752	14,077	40.4	25.8	23.8	10	66.2	33.8	-0.411	-0.639
Trichinellida: Trichuridae										
* Trichuris ovis*	NC_018597	13,946	34.5	35.3	15.9	14.4	69.8	30.3	-0.05	0.01
* Trichuris discolor*	NC_018596	13,904	33.9	36	15.3	14.9	69.9	30.2	-0.012	-0.033
* Trichuris muris*	NC_028621	14,105	35.6	37.8	12.7	13.8	73.4	26.5	0.043	-0.089
* Trichuris muris*	AP017703	14,297	35.5	36.4	14.7	13.5	71.9	28.2	-0.043	-0.131
* Trichuris suis*	NC_017747	14,436	35.6	35.9	15.1	13.5	71.5	28.6	-0.057	-0.165
* Trichuris trichiura*	NC_017750	14,046	33.6	34.5	16.9	15	68.1	31.9	-0.059	-0.263
* Trichuris rhinopiptheroxella*	MG189593	14,186	33.4	36	15.4	15.2	69.4	30.6	-0.007	-0.315
* Trichuris sp.*	KT449824	13,984	34.2	35	16.5	14.3	69.2	30.8	-0.07	-0.537

All base composition values are shown for the mitochondrial plus strand. The ‘length’ refers to the full length of the mitogenome in bases. Base composition is given in %. GC means GC skew, and GC_NCR is GC skew of all noncoding regions

This is indicative of inversions in the strand replication order, or otherwise disrupted replication mechanism (e.g. multiple origins of replication) in the lineages with reduced/inverted skews [16, 22]. As skews were not discussed in previous papers that reported clade I mitogenomes with inverted skews [6–8, 43–45], this is the first observation that some enoplean lineages underwent skew inversions. To get a better resolution we checked all other available Nematoda mitogenomes. This revealed that all chromadorean mitogenomes exhibit high positive GC skews on the plus strand: 0.18 to 0.64 (average = 0.40) (Additional file 2: panel D). As discussed above, Trichinellidae possess the most ancestral architecture among all nematodes, whereas all other lineages exhibit highly derived architectures. From this, we can infer that a high negative GC skew on the plus strand, similar to the one exhibited by most other Arthropoda [16, 46], is the ancestral skew for Nematodes. Therefore, chromadorean nematodes underwent a series of architectural rearrangements that resulted in all genes being encoded on a single strand and resulted in a full skew inversion on the plus strand (alternatively, but less likely, all genes from the plus strand may have migrated to the minus strand).

Theoretically, it may be possible to identify strand inversions of the origin of replication from conserved promoter sequence motifs [16]. For example, in *Xiphinema americanum*, a sequence motif 5’-GAGACCTGAGCCCAAGATA-3’ similar to the conserved promoter element sequence in the human mitogenome was found in the putative CR [5]. We assessed our dataset for the presence of this motif, but it matched to (highly derived) sequences in only four species: two *Xiphinema* and two Mermithidae species (Additional file 1: Figure S15). We also searched for other conserved motifs but, apart from several motifs that are common in Trichinellidae, other families did not exhibit any consistently conserved motifs (Additional file 3). This is in agreement with a previous study, which found no identifiable conserved motifs in Longidoridae [6]. Several common motifs were identified in the Mermithidae, but their apparent abundance was largely attributable to the multiplication of NCRs in this family. This further corroborates the fast and unique evolution of mitochondrial control regions in the Enoplea.

### Phylogenetic analyses and implications of inverted skews for evolutionary analyses

**Fig. 2:**
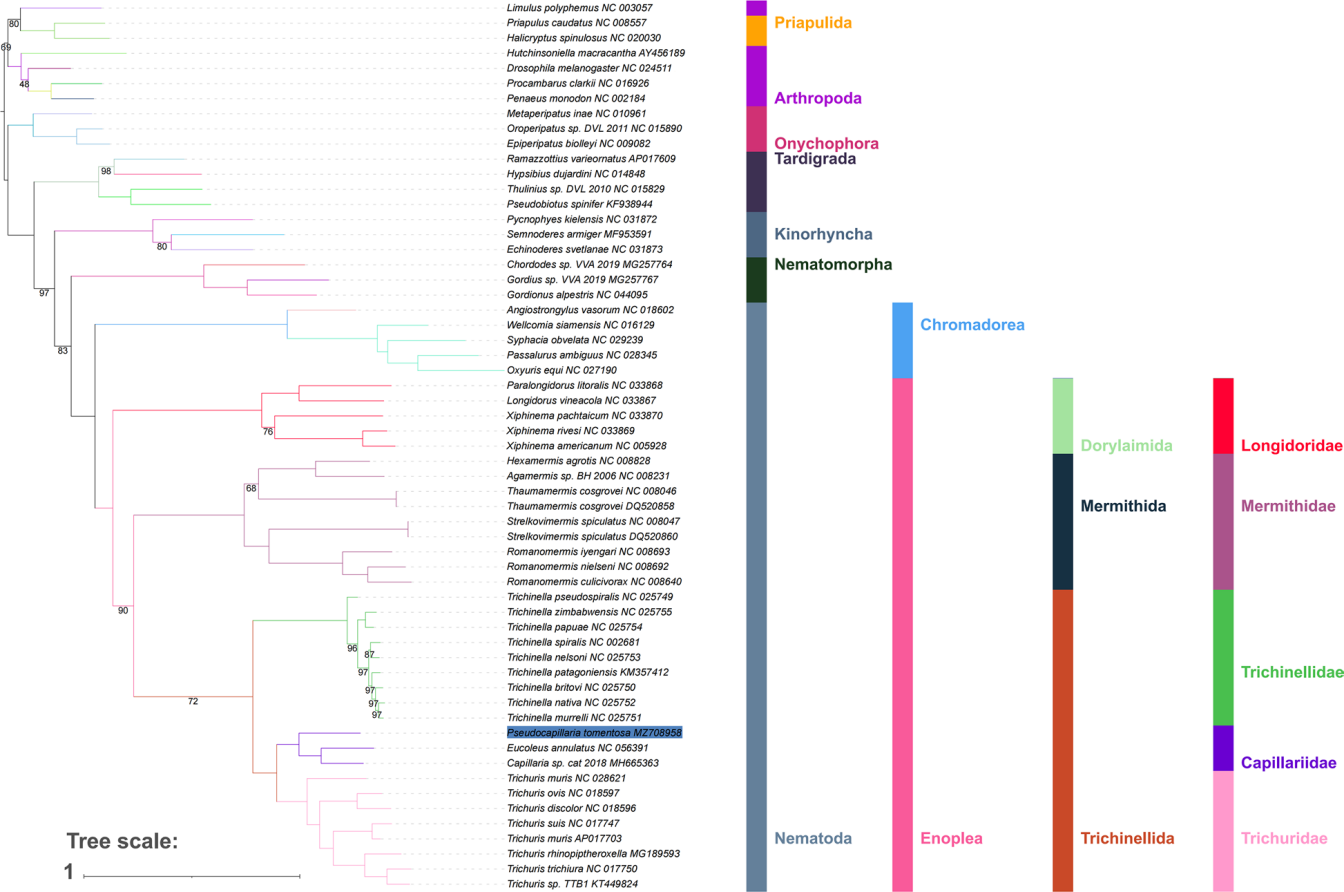
Mitochondrial phylogenomics of the Enoplea: Maximum Likelihood. The phylogram was inferred using the Maximum Likelihood methodology implemented in IQ-TREE and nucleotide sequences of 12 protein-coding genes. Bootstrap support values are shown on the braches (only the values < 100 are shown). Species names are given with GenBank accession numbers. The phylum, class, order, and family-level taxonomic identities are shown to the right

**Fig. 3:**
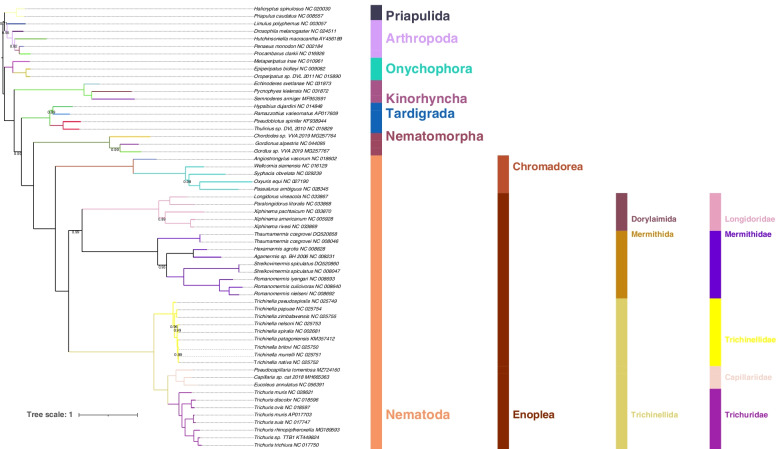
Mitochondrial phylogenomics of the Enoplea: CAT-GTR. The phylogram was inferred using the CAT-GTR algorithm designed for compositional heterogeneity implemented in PhyloBayes and amino acid sequences of 12 protein-coding genes. Posterior probability values are shown on the braches (only the values < 1.0 are shown). Species names are given with GenBank accession numbers. The phylum, class, order, and family-level taxonomic identities are shown to the right

The phylogeny of Enoplea remains unresolved, as different datasets produce incongruent results. Previous evidence that skew inversions affect phylogenetics and other evolutionary studies was limited to arthropods (mostly crustaceans), which often exhibit fully inverted skew biases (i.e. almost equal magnitude in opposite direction; e.g. -0.25 and + 0.25) [14, 16, 17]. However, skew inversion in enoplean nematodes is only partial, so effects on evolutionary analyses may be less strongly pronounced. We found that only two sequences (*Capillaria sp.* and *T. muris*) passed the compositional homogeneity test. Mutational saturation analyses indicated substantial or high levels of saturation (defined as transitions and transversions plateauing) for most individual genes and for the 3^rd^ codon position dataset. However, concatenated 12 PCGs dataset and 1^st^ and 2^nd^ codon datasets did not exhibit strong levels of saturation (Additional file 1: Fig. S16). Also, ORI species did not appear to exhibit elevated levels of saturation. This indicates that concatenated mitogenomic datasets in combination with an algorithm that accounts for compositional heterogeneity may be a suitable tool for studying the evolutionary history of the Enoplea. However, previous (recent) studies that studied the enoplean mitochondrial phylogenomics relied on standard phylogenetic analysis algorithms: Maximum Likelihood (ML), Bayesian Inference (BI), and Maximum Parsimony (MP) [6, 12, 13]. Herein we tested the performance of the standard ML phylogenetic approach implemented in IQ-TREE, as well as the CAT-GTR algorithm designed to account for compositional heterogeneity [47]. The ML analysis produced paraphyletic Arthropoda, with Priapulida nested within, and a generally non-standard [48] topology of Ecdysozoa, but Nematoda and Nematomorpha were resolved as sister-phyla, which is accepted as the most likely evolutionary scenario [49]. Chromadorea and Enoplea were monophyletic sister-taxa, and Longidoridae were the basal Dorylaimia lineage (i.e. sister-clade to all remaining lineages) (Fig. 2). Mermithida and Trichinellida were sister-groups, and Capillariidae + Trichuridae exhibited a close sister-clade relationship in the latter clade (Fig. 2). In all crucial aspects, the tree produced using all available genes was identical (Additional file 4). The CAT-GTR model produced a similar topology, but with monophyletic Arthropoda (sister-group relationship with Priapulida), switched places between Kinorhyncha and Tardigrada, and with the Enoplean topology resolved into two major sister-clades: Dorylaimida + Mermithida and Trichinellida (Fig. 3). Support values for key nodes were higher in the CAT-GTR analysis than in the ML analysis, where Trichinellidae + (Mermithidae + Capillariidae) clade was weakly supported: 72% (100% in the CAT-GTR).

With respect to the enoplean topology, *18S* datasets appear to resolve Enoplia and Dorylaimia as sister clades, often with Trichinellidae and/or Dioctophymatida as the basal Dorylaimia clade, but results are inconsistent or topologies unresolved [1, 6, 50, 51]. The available nuclear genomic data resolve Mermithidae as the basal lineage [52], but many crucial lineages remain unrepresented. The available mitogenomic data mostly produce two different topologies across different studies: 1. Longidoridae as the basal clade, and 2. Enoplea comprised of two sister-clades: Dorylaimida + Mermithida and Trichinellida. Topology 1 was produced by the nucleotide dataset (NUC) in our ML analysis with high support (100%), and by two previous studies: ML AAs analysis (amino acids) [13], and ML and BI AAs [6]. Topology 2 was produced by our CAT-GTR analysis (100% support; AAs dataset), and by several previous studies: BI and ML AAs [12], BI AAs [13], BI and ML NUC [6], and ML, BI, and MP AAs [41]. The topology of the remaining enoplean lineages is remarkably consistent across all studies. In comparison, the available genomic data produce Mermithidae as the basal radiation, followed by the Dioctophymatida and finally Trichinellidae and Trichuridae as highly derived sister-families, but the absence of data for Longidoridae remains a major shortcoming [52].

This uncertainty also makes it difficult to infer the evolutionary history of replication disruption events (producing changes in skew magnitude) in the enoplean nematodes. If Trichniellidae is the earliest-branching lineage, which is unlikely as it is partially supported only by the *18S* data, and consistently rejected by mitogenomic and nuclear genomic data, a single disruption of the replication mechanism is sufficient to explain the observed pattern. The topology produced by the mitogenomic data requires a more complex evolutionary scenario. Topology 1 would require three independent disruptions: in the common ancestors of Longidoridae, Mermithidae, and Capillariidae + Trichuridae. Topology 2 would require two independent disruptions: in the common ancestors of Longidoridae + Mermithidae and Capillariidae + Trichuridae. Therefore, topology 2 is supported by the more reliable CAT-GTR analysis in our study, a majority of previous mitogenomic results, and by being the more parsimonious of the latter two skew disruption scenarios.

## Conclusions

To improve our understanding of the discontinuity in the evolution of mitogenomes in the nematode class Enoplea, we sequenced and characterised the complete mitochondrial genome of *P. tomentosa* (Trichinellida: Capillariidae). As mitochondrial molecular data were previously unavailable for this species, the sequence will facilitate future molecular identification and evolutionary studies of this species. Similar to the Chromadorea, the evolution of mitochondrial architecture in the enoplean nematodes exhibits a strong discontinuity: lineages with relatively conserved architecture over tens of millions of years are interspersed with lineages exhibiting architectural hypervariability. Surprisingly, Longidoridae exhibited more highly rearranged mitogenomes than Mermithidae, which possess some of the fastest-evolving mitochondrial architecture among the Bilateria. This is in contradiction with the expectation that high rearrangement rates should produce multiple noncoding regions in mitogenomes. We provide the first observation of an inverted base composition skew in the enoplean lineages. Lineages exhibiting inverted skews appear to represent the intermediate phase between the Trichinellidae (ancestral) and fully derived (high positive) skews in Chromadorean mitogenomes. Our observations have important repercussions for future studies that aim to apply mitochondrial phylogenomics to nematodes. Despite the inverted skews and overall compositional heterogeneity, we found evidence that mitochondrial and nuclear phylogenomics might produce congruent topologies. In the absence of Enoplia and Mononchida representatives, multiple lines of evidence (CAT-GTR analysis in our study, a majority of previous mitogenomic results, and skew disruption scenarios) support the Dorlylaimia split into two sister-clades: Dorylaimida + Mermithida and Trichinellida. However, it is necessary to sequence data from the missing crucial lineages before this can be assessed with confidence, and future studies should pay close attention to putative artefacts caused by compositional heterogeneity and seek agreement between different types of data before any conclusions are made.

## Methods

### Sample collection and identification

Parasitic nematodes were obtained post mortem from bighead carp *Hypophthalmichthys nobilis* specimens caught by fishermen in the Bailianhe reservoir (Huanggang city, Hubei province, China) and bought from the local market on 2/Aug/2020. Live nematodes were removed from the fish intestines, and then taxonomically identified by their morphological characteristics [24] via dissecting microscopy. All nematodes were washed in 0.6% saline before being stored in the absolute ethanol in the Museum of Aquatic Organisms, Institute of Hydrobiology, Chinese Academy of Sciences, Wuhan, China. Further identification was conducted using the *18S* gene sequence, for which primers (Table 4) were designed on the basis of the previously sequenced conspecific *18S* sequence KU987805 [23].

**Table 4:** Primers used for the amplification and sequencing of the mitochondrial genome of ***P. tomentosa***. LR next to the fragment number means that long-range PCR was used to obtain the amplicon. See Additional file 1 for the full list of sequencing primers

Fragment No	Gene or region	Primer name	Sequence (5’-3’)	Length (bp)
F1	*16S*	Eno 16SF	GTTTKTGACCTCGATGTTGN	171
		Eno 16SR	CYTTTWGTTCCTTTCGTACT	
F2 (LR)	*16S-cox1*	MX F1	AACGTCTGTTCGACGTAAGA	3395
		MX R1	CTACATCCATACCTACGGTG	
F3	*cox1*	Eno COX1F	GATTHTTNGGTCAYCCTGAAGT	614
		Eno COX1R	ATACCGWCGNGGTATACCAT	
F4 (LR)	*cox1-tRNA-Thr*	MX F2	GATTGCCATGAATGATAGGA	7150
		MX R2	CAAAATCTATATTCTACTTAAAC	
F5 (LR)	*nadL-16S*	MX F3	CAAAACCAATAATTCTGTGTG	3469
		MX R3	TCTTACGTCGAACAGACGTT	

### Genome sequencing and assembly

Genome sequencing and assembly were conducted as described before [9], so details are provided in Additional file 1. Briefly, DNA was isolated from a single specimen. Five primer pairs were designed on the basis of gene orthologues from *Capillaria* sp., and then used to amplify the entire mitogenome (Table 4). To avoid assembly artefacts, the amplified fragments were designed to overlap by approximately 100 bp. PCR products were sequenced using the Sanger method and an expanded set of primers, because fragments longer than 1kbp (amplified using long-range PCR) had to be sequenced in several steps (see Additional file 1 for full details). The mitogenome was assembled manually using DNAstar v7.1 [53], and roughly annotated using MITOS [54]. The annotation was further refined using several different methods: DNAstar, BLAST BLASTx (PCGs), orthologous sequences (PCGs and rRNAs), and ARWEN [55] (tRNAs).

### Comparative mitogenomic and phylogenetic analyses

We downloaded all available Enoplea mitogenomes from GenBank. We removed all duplicates, two unannotated mitogenomes, and left only one mitogenome per species (unless we found indications that conspecific mitogenomes might exhibit a different architecture). PhyloSuite [56] was used to conduct these steps, as well as to parse and extract the annotation recorded in a Word (Microsoft Office) document, generate the GenBank format file, update the taxonomy from the NCBI database, standardise and extract data, generate comparative tables, and generate annotation files for visualisation in iTOL [57]. GC and AT base composition skews were also calculated by PhyloSuite, following the (G-C)/(G + C) and (A-T)/(A + T) formulas respectively [42]. PhyloSuite and its plug-in programs were also used to conduct all phylogenetic analysis steps, for which we used nucleotide sequences of concatenated 12 mitochondrial protein-coding genes (PCGs); *atp8* was removed because it was absent from many species, and only one copy per gene was kept in the Mermithidae that exhibited duplicated PCGs (further details in Additional file 1). Sequence alignment in batches was conducted using the accurate E-ins-i strategy in MAFFT [58]. Alignments were concatenated by Phylosuite. Phylogenetic analyses were conducted using IQ-TREE (Maximum Likelihood – ML) [59] and the CAT-GTR site mixture model implemented in PhyloBayes-MPI 1.7a [47], which allows for site-specific rates of mutation, as there is evidence that in some cases it alleviates the base composition skew-driven long-branch attraction artefacts better than other standard phylogenetic algorithms, especially in combination with amino acid sequences [17]. PhyloBayes run parameters were burnin = 500, invariable sites automatically removed from the alignment, two MCMC chains, and the analysis was stopped when the conditions considered to indicate a good run were reached: maxdiff < 0.1 and minimum effective size > 300 (PhyloBayes manual). The best-fit partitioning strategy and models for partitions for IQ-TREE were inferred using the inbuilt functions of IQ-TREE and immediately followed by phylogenetic reconstruction with 50,000 ultrafast bootstrap replicates [60]. We also conducted an IQ-TREE analysis using the parameters described above to test whether the inclusion of all genes (PCGs + rRNAs + tRNAs) can stabilise the topology. IQ-TREE was also used to test for compositional heterogeneity. For mutational saturation analyses [61] and to generate cumulative skew plots for the entire plus strand, we used DAMBE 7.3.0 [62]. CREx was used to infer the GO distances [29]. All GenBank files were rearranged to start with the *cox1* gene using the *Reorder* function in PhyloSuite, which was also used to generate the comparative mitogenomic architecture table for the *P. tomentosa* and *E. annulatus* mitogenomes. Conserved sequence motifs searches were conducted using MEME [63] and FIMO [64] tools.

## Supplementary Information

**Additional file 1.**

**Additional file 2.** 

**Additional file 3.**

**Additional file 4.** 

## Data Availability

The datasets supporting the conclusions of this article are included within the article and its additional files, as well as in the GenBank repository. The complete mitogenome of *P. tomentosa* is available under the accession number MZ708958 (https://www.ncbi.nlm.nih.gov/nuccore/MZ708958.1/) and the partial *18S* sequence is available under MZ724160 (https://www.ncbi.nlm.nih.gov/nuccore/MZ724160).

## Abbreviations

AAs: Amino acids
Bp: Base pair
CR: Control region
ML: Maximum likelihood
MP: Maximum parsimony
BI: Bayesian inference
NCR: Noncoding region
NUC: Nucleotides
PCGs: Protein-coding genes
