# Supplementary material for: Inverted base composition skews and discontinuous mitochondrial genome architecture evolution in the Enoplea (Nematoda)
Source: BMC Genomics. 2022 May 18;23:376. doi: 10.1186/s12864-022-08607-4 (PMC9115964; doi:10.1186/s12864-022-08607-4)
Supplement: Supplementary file 1 — Additional file 1. [file 12864_2022_8607_MOESM1_ESM.pdf]

## Additional file 1. Additional Results, Discussion, and Methods

### Inverted base composition skews in the mitochondrial genomes of Enoplea (Nematoda)

Hong Zou, Fang-Lin Chen, Wen-Xiang Li, Ming Li, Hong-Peng Lei, Dong Zhang, Ivan Jakovlić, Gui-Tang Wang

* Corresponding authors, E-mail addresses: ivanjakovlic@yahoo.com (IJ) and gtwang@ihb.ac.cn (GTW); Address: Key Laboratory of Aquaculture Disease Control, Ministry of Agriculture, Institute of Hydrobiology, Chinese Academy of Sciences, Wuhan 430072, China.

## Table of Contents

Additional Results and Discussion ........ 2
Species identity ........ 2
Figure S1. Phylogenetic analysis (ML) of all 245 *18S* sequences available for the Capillariidae. ... 3
Figure S2. Phylogenetic analysis (ML) of 75 Capillariidae 18S sequences. ........ 4
General features of the enoplean mitogenomes ........ 4
Figure S3. The mitochondrial architecture for all available Trichinellidae mitogenomes. ........ 6
Gene order ........ 7
Figure S4. Gene order rearrangements. ........ 7
Figure S5. Gene order rearrangements. ........ 8
Figure S6. Gene order rearrangements. ........ 9
Figure S7. Gene order rearrangements. ........ 10
Base composition and skews ........ 11
Figure S8. Cumulative GC skew plot for the *Pseudocapillaria tomentosa* mitogenome. ........ 11
Figure S9. Cumulative GC skew plot for the *Capillaria* sp. mitogenome. ........ 12
Figure S10. Cumulative GC skew plot for the *Eucoleus annulatus* mitogenome. ........ 12
Figure S11. Cumulative GC skew plot for the *Romanomermis culicivorax* mitogenome. ........ 13
Figure S12. Cumulative GC skew plot for the Strelkovimermis spiculatus mitogenome. ........ 13
Figure S13. Cumulative GC skew plot for the *Xiphinema americanum* mitogenome. ........ 14
Figure S14. Cumulative GC skew plot for the Xiphinema pachtaicum mitogenome. ........ 14
Figure S15. Cumulative GC skew plot for the *Trichuris muris* mitogenome. ........ 15
Figure S16. Cumulative GC skew plot for the *Trichuris suis* mitogenome. ........ 15

Figure S17. Cumulative GC skew plot for the *Trichinella spiralis* mitogenome. .......................... 16
Figure S18. Cumulative GC skew plot for the *Trichinella nativa* mitogenome. .......................... 16
Figure S19. FIMO search for the sequence motif 5′-GAGACCTGAGCCCAAGATA-3′ .............. 16
Figure S20. Mutation saturation analyses for individual genes, concatenated genes (PCGs), and codon positions. .......................... 17
Additional Methods .......................... 17
Genome sequencing and assembly .......................... 17
Phylogenetic analyses .......................... 19
References .......................... 20

## Additional Results and Discussion

### Species identity

We first identified the collected specimens morphologically [1], but morphological identification of nematodes is notoriously difficult and error-prone [2], and *cox1* barcode sequences remained unavailable until we sequenced this mitogenome, so we also sequenced a 1,171 bases-long fragment of the *18S* rRNA gene. *Cox1* exhibited the highest similarity to the *Capillaria* sp. *cox1* sequence (82.2%). This is relatively low for *cox1* sequences belonging to species from the same family [3], but it can be explained by the fact that in nematodes *cox1* evolves faster than in most other metazoan lineages [4]. The 18S sequence exhibited a 100% identity (100% coverage) with the only available *P. tomentosa 18S* sequence: KU987805. This is intriguing as the latter parasite was collected from *Cyprinella lutrensis* in North America [5], so we expected it to be genetically distant from Chinese populations. While global trade has caused admixture of many previously isolated populations, the high similarity is most likely to be attributed to the fact that *18S* is generally highly conserved in nematodes, so it has limited applicability for distinguishing closely related species [2, 6]. Importantly, a recent study found that *18s* performed very well in Capillariidae and that it exhibited perfect reliability in the identification of genera and very good reliability in the identification of species [7]. We conducted two Maximum Likelihood (ML) phylogenetic analyses. The first one was conducted on the complete Capillariidae *18S* dataset (245 sequences; Figure S1). Due to sequences covering different segments of the *18S* gene and often exhibiting very small overlap with other sequences, this analysis produced a rather noisy overall result, with many paraphyletic genera and species, but the new sequence formed a monophyletic clade with the only available *Pseudocapillaria* sequence

(KU987805). Following this, we pruned the dataset to 75 sequences in a way to maximise the phylogenetic signal, i.e. ensure maximum sequence coverage with the newly sequenced *18S* fragment. This stabilised the topology, so most genera and species were monophyletic (with a few exceptions), and the new sequence also formed a distinct monophyletic clade with the only available *Pseudocapillaria* sequence (Figure S2). Therefore, molecular data place the sequenced specimen into the *Pseudocapillaria* genus with full confidence.

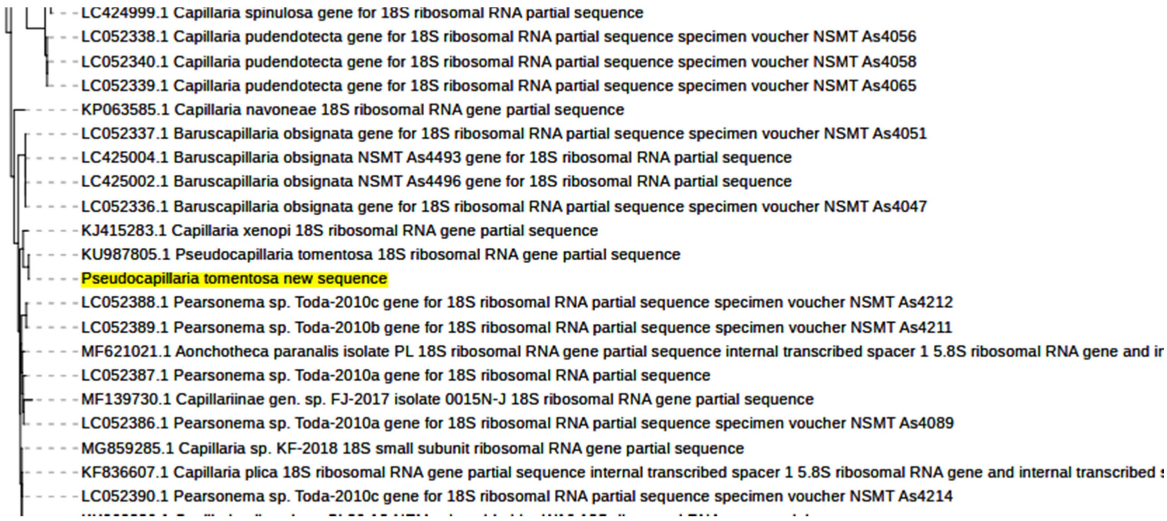


**Figure S1. Phylogenetic analysis (ML) of all 245 *18S* sequences available for the Capillariidae.** Only a fragment of the phylogram is shown. The *18S* fragment sequenced for this study (*P. tomentosa*) is highlighted in yellow.

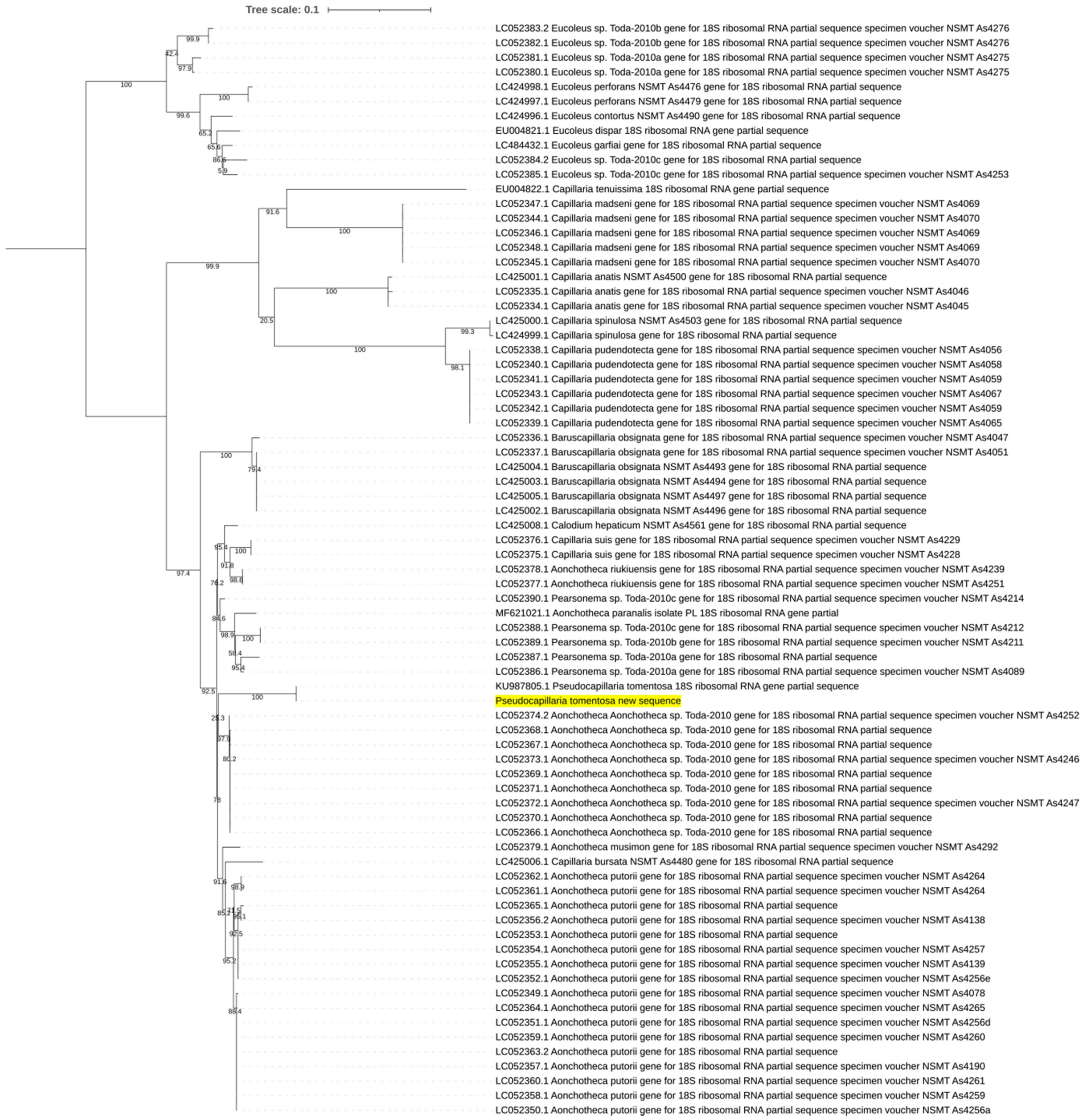


**Figure S2. Phylogenetic analysis (ML) of 75 Capillariidae 18S sequences.** These sequences exhibited almost full sequence coverage with the *18S* fragment of *P. tomentosa* sequenced for this study (highlighted in yellow).

## General features of the enoplean mitogenomes

The mitogenome of *Capillaria* sp. possessed numerous unique features strongly indicative of sequencing artefacts, most notably reduced overall size (by approx.. 400 bp), truncated PCGs, and five missing tRNA genes (Additional file 2: 'Gene info' panel), so we did not include it in detailed comparative analyses. PCG sizes in *P. tomentosa* were in the standard range for Trichinellida. A

minor exception was *nad4*, which was a bit larger than in *Capillaria* sp. (1260 vs. 1218) due to and insertion at the 5’ end of the gene, but we could not identify any signs of annotation or sequencing errors. We should note that *E. annulatus nad4* was much shorter due to a long deletion at the 5’ end, and *nad2* of *Capillaria* sp. was almost 300 bp shorter than in the other two Capillariidae species. Both of these are almost certainly artefacts, so we can infer with relative confidence that both other Capillariidae mitogenomes are either incomplete or improperly annotated. Among the four intergenic regions ≥40bp identified in *P. tomentosa*, two of them were conserved with *E. annulatus*: 98/45 bp, respectively, between *trnC* and *trnQ*, and 50/55 bp, respectively, between *nad2* and *trnM*.

Most Trichinellidae species possessed at least one standard-sized (>500 bp) NCR. Exceptions were *Trichinella zimbabwensis* and *Trichinella nativa*, which do not appear to possess a large NCR. As sequencing of mitochondrial noncoding regions is often very difficult due to their complex secondary structure and repetitive elements, we checked all available Trichinellidae mitogenomes to assess whether these may be annotation artefacts (Figure S3). Among the two available *T. nativa* mitogenomes (NC_025752 and KM357415 are identical), the AP017702 species exhibits a single 1260 bp NCR in the conserved position between *trnK* and *nad2*. This confirms a sequencing artefact in this species. As there is only one mitogenome available for *Trichinella zimbabwensis* (the two in the figure are GenBank and RefSeq versions of the same mitogenome), we cannot confirm sequencing artefacts with confidence, but the fact that it possesses three NCRs >100 bp, including one between *nad1* and *nad2,* it is likely that the mitogenome may be incomplete. Similarly, *Trichuris rhinopiptheroxella* had an NCR of 794 bp between *rrnL* and *cox3*, but that is most probably the unannotated *atp6* gene (see Figure 1, main manuscript).

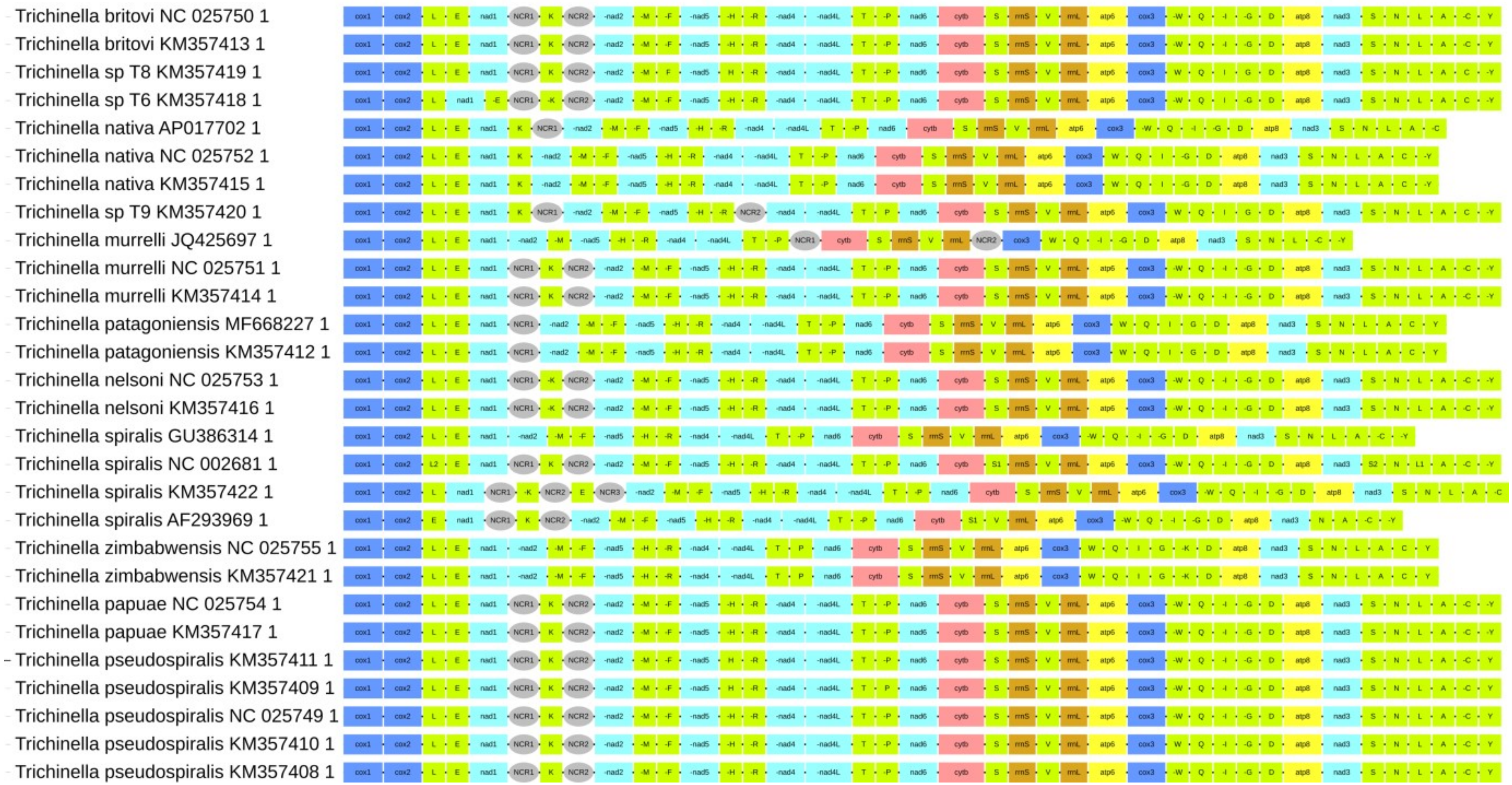


**Figure S3. The mitochondrial architecture for all available Trichinellidae mitogenomes.** Only NCRs ≥ 200 bp are shown.

## Gene order

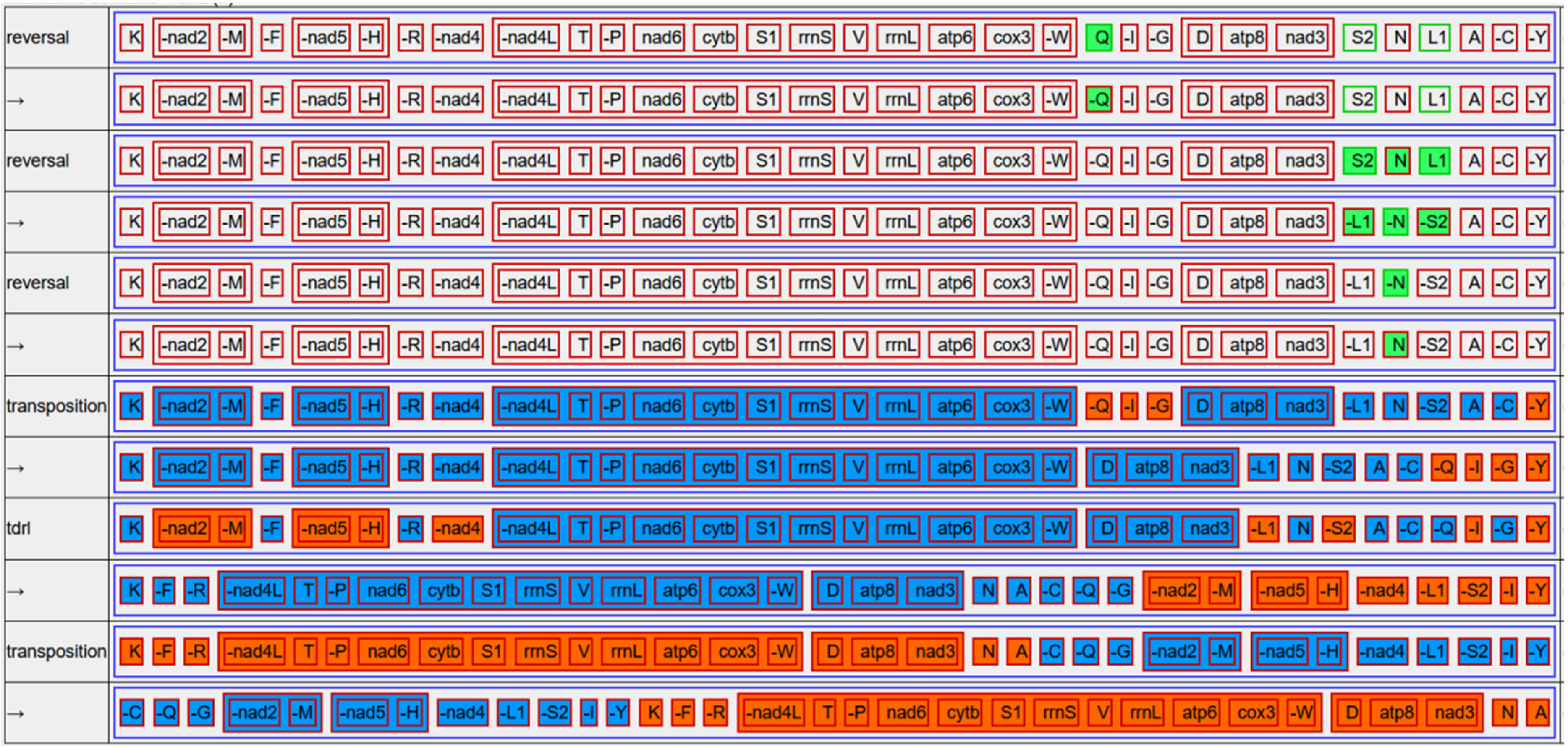


**Figure S4. Gene order rearrangements.** Putative gene order rearrangement scenario from the gene order exhibited by *Pseudoapillaria tomentosa* to the ancestral mitogenomic order exhibited by most Trichinelidae (*Trichinella_papuae, Trichinella_murrelli, Trichuris_muris, Trichuris_trichiura, Trichuris_suis, Trichuris_rhinopiptheroxella, Trichuris_sp, Trichuris_muris, Trichinella_spiralis*) inferred using CREX. tdrl stands for tandem duplication/random loss. Only one out of seven possible scenarios is shown.

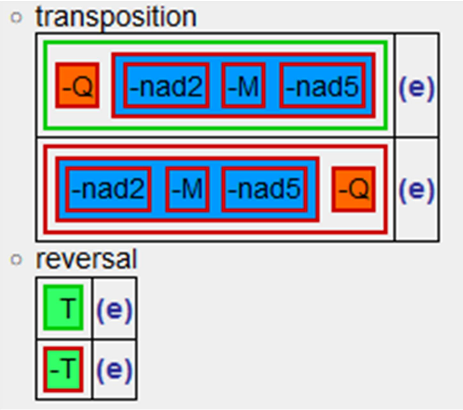


**Figure S5. Gene order rearrangements.** Gene order rearrangement scenario from the gene order exhibited by *Pseudoapillaria tomentosa* to that of *Capillaria* sp. See Figure S4 for other details.

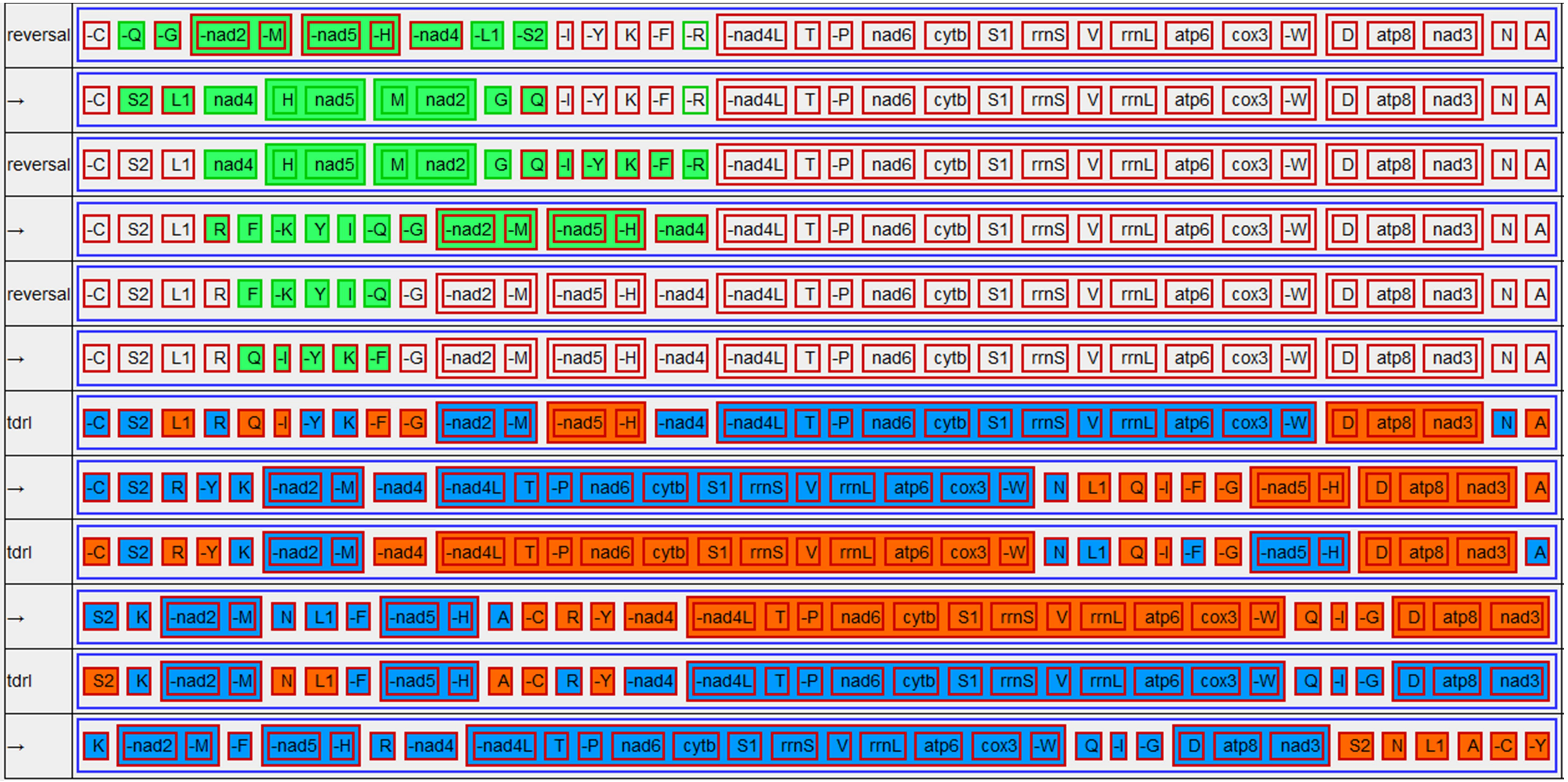


**Figure S6. Gene order rearrangements.** Gene order rearrangement scenario from the ancestral mitogenomic order of *Pseudoapillaria tomentosa* to that of *Trichuris discolor*. Only one out of three possible scenarios is shown. See Figure S4 for other details.

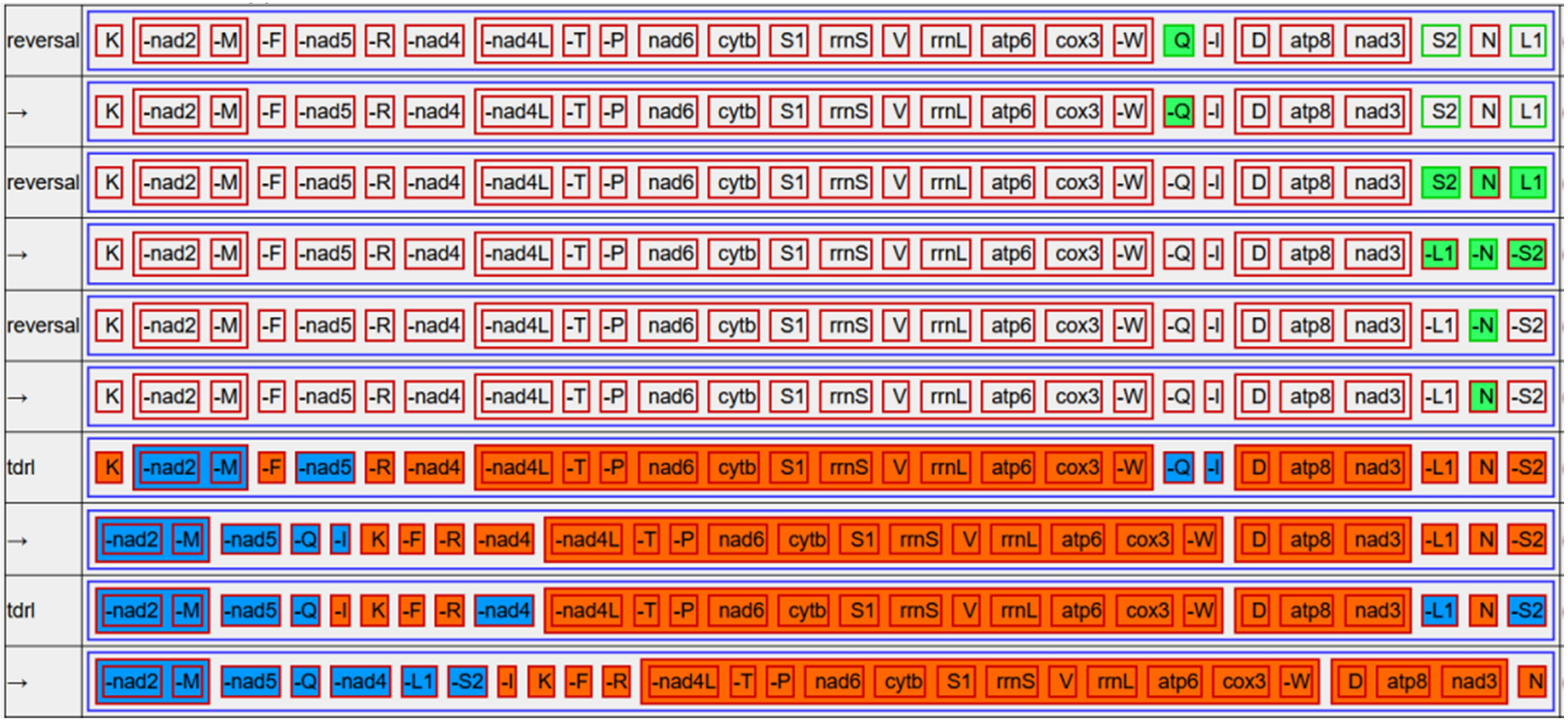


**Figure S7. Gene order rearrangements.** Gene order rearrangement scenario from the ancestral mitogenomic order of Trichinelidae to the one exhibited by *Capillaria* sp. Only one out of four possible scenarios is shown. See Figure S4 for other details.

## Base composition and skews

In the dataset, comprising all Nematoda mitogenomes available in the curated RefSeq database (Nematoda174 dataset, panel C in the Additional file 2) [8], negative GC skews on the plus strand were exhibited by five Chromadorea species: *Caenorhabditis brigsae* and four *Litoditis aff. marina* mitogenomes. However, all five of them had all genes encoded on the minus strand. This confirms that the authors submitted a 'wrong' mitochondrial strand (minus strand) to the GenBank and that these five species possess standard mitochondrial skews for Chromadorea (0.24 to 0.26).

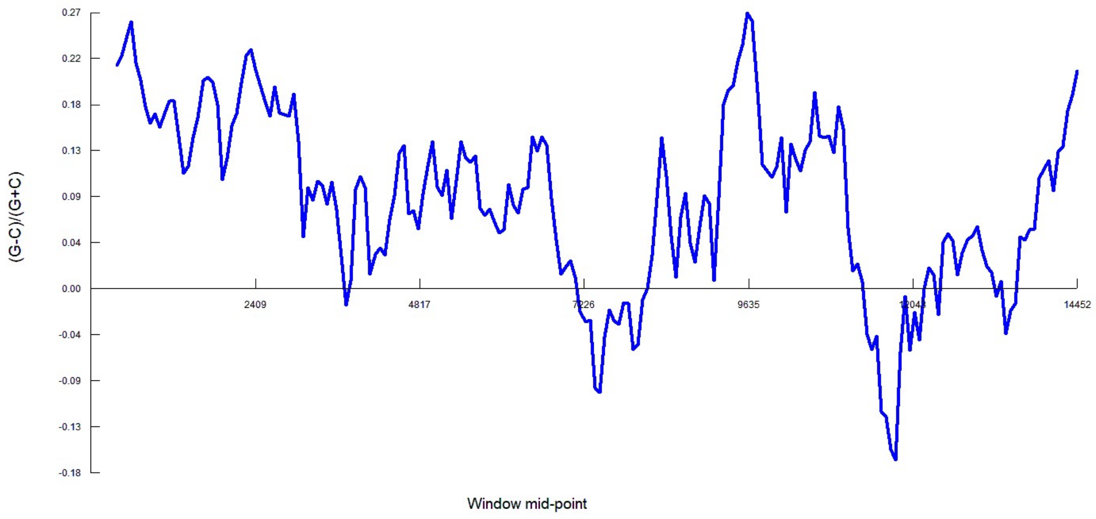


**Figure S8. Cumulative GC skew plot for the *Pseudocapillaria tomentosa* mitogenome.** The x-axis shows the mitogenome and y-axis cumulative skews. The optimal window and step sizes were inferred automatically according to GC skewness using DAMBE.

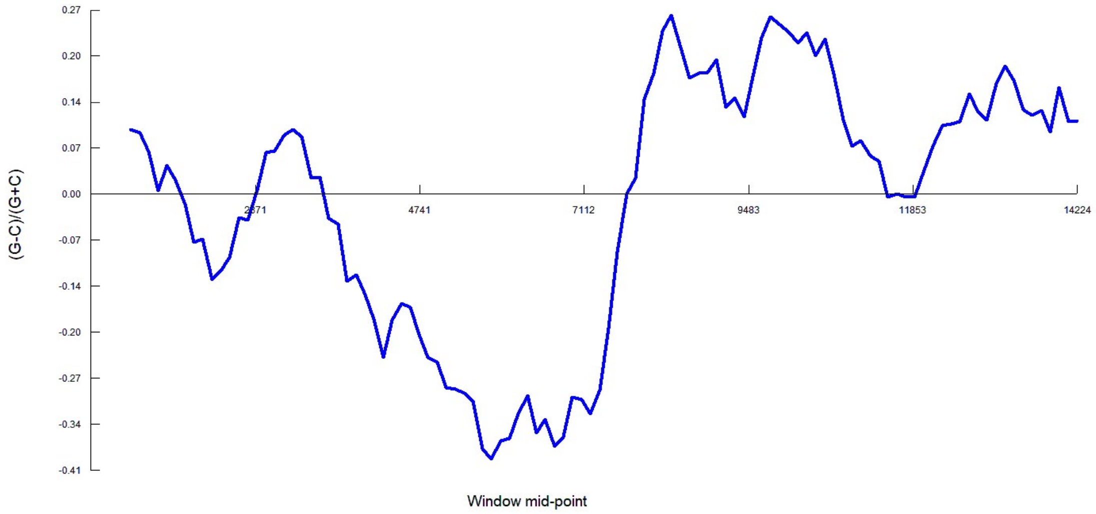


**Figure S9. Cumulative GC skew plot for the *Capillaria* sp. mitogenome.**

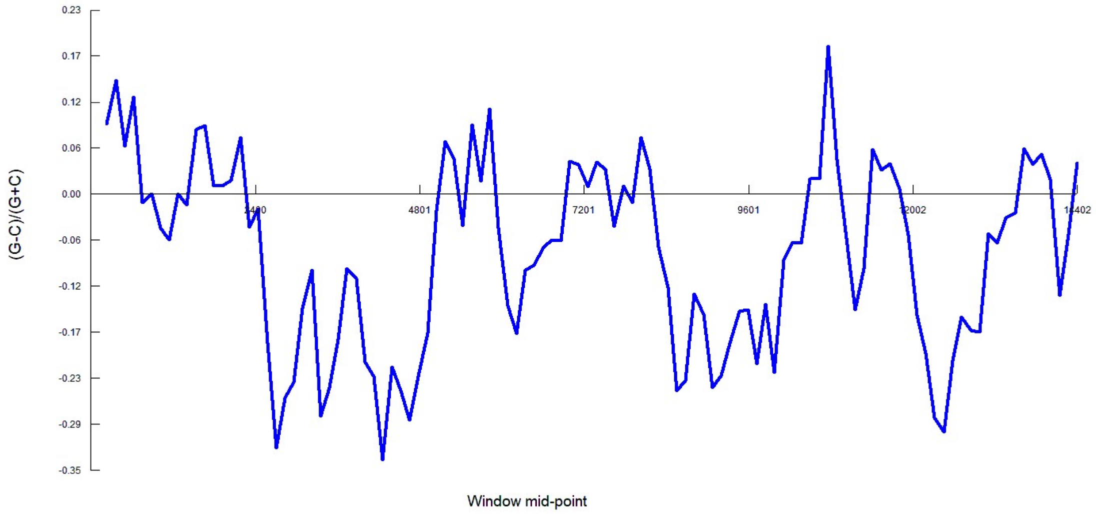


**Figure S10. Cumulative GC skew plot for the *Eucoleus annulatus* mitogenome.**

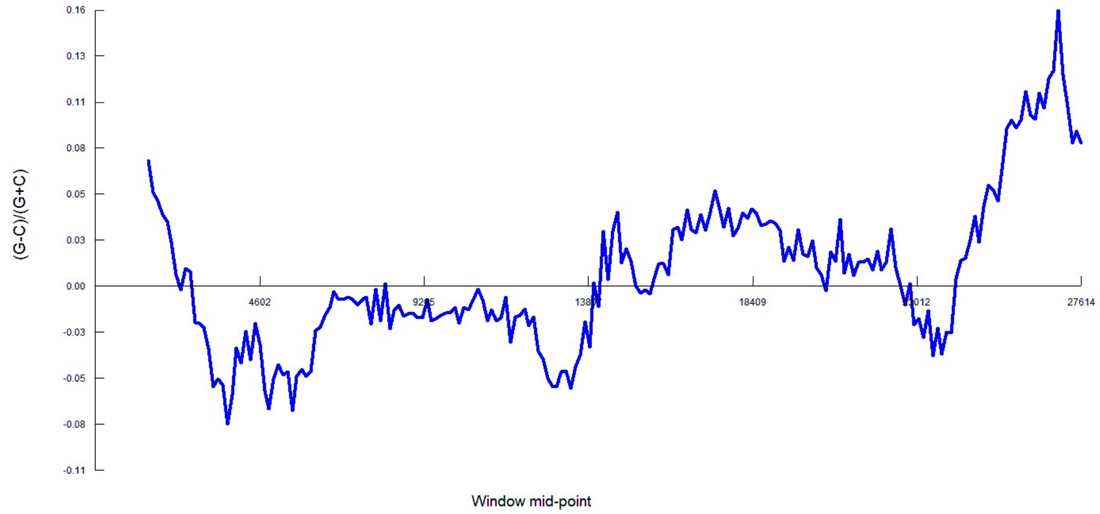


**Figure S11**. **Cumulative GC skew plot for the *Romanomermis culicivorax* mitogenome.**

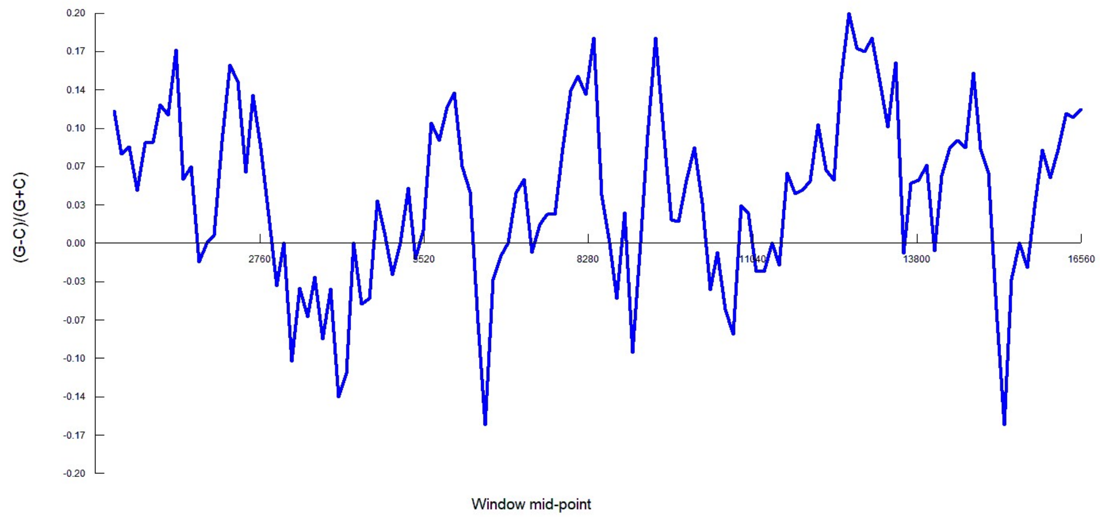


**Figure S12. Cumulative GC skew plot for the *Strelkovimermis spiculatus* mitogenome.**

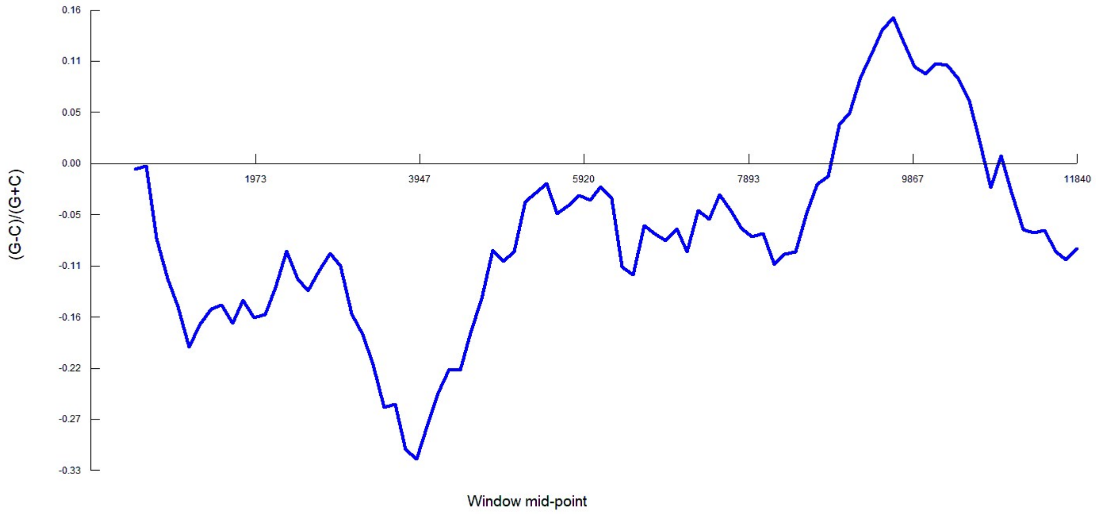


**Figure S13**. **Cumulative GC skew plot for the *Xiphinema americanum* mitogenome.**

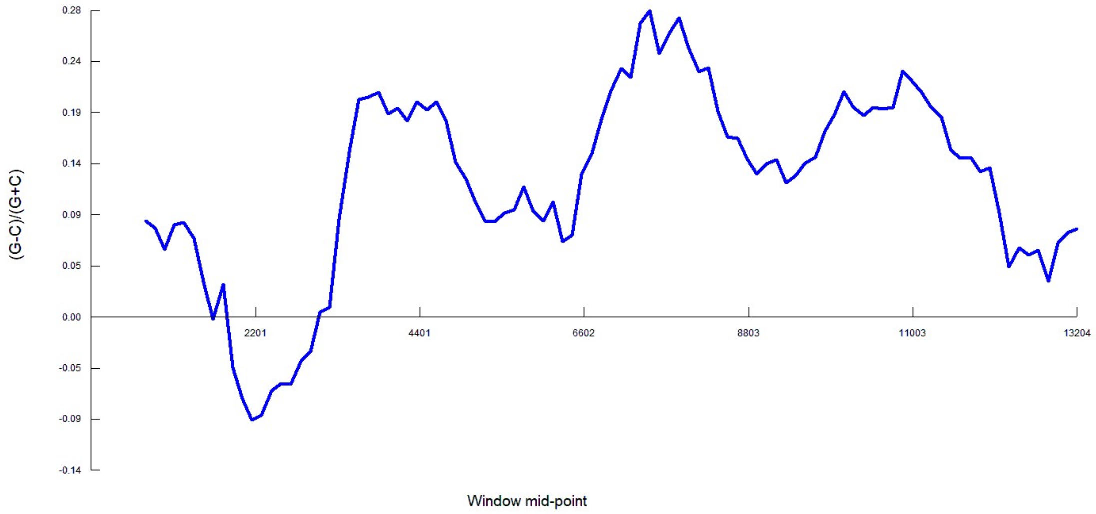


**Figure S14. Cumulative GC skew plot for the Xiphinema pachtaicum mitogenome.**

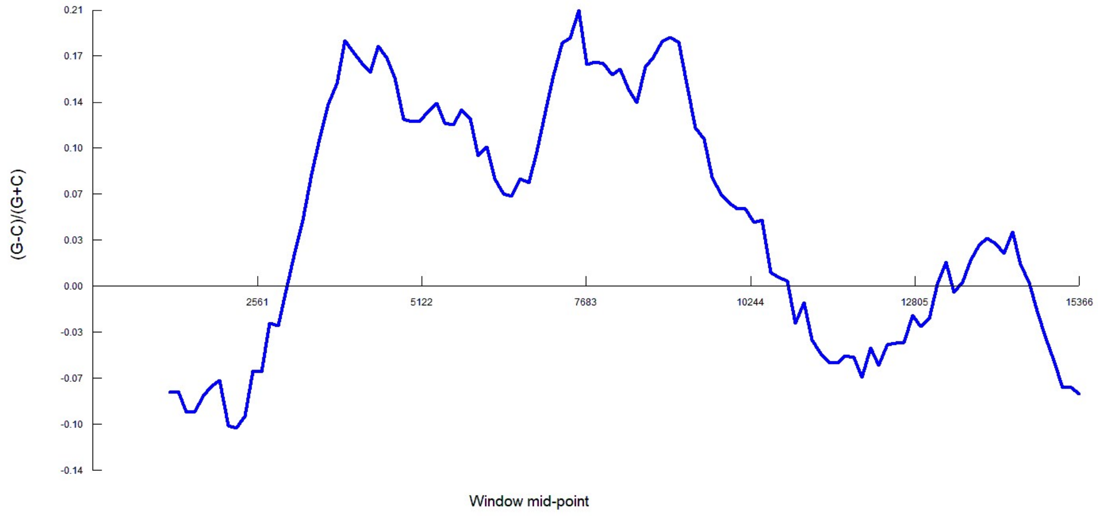


**Figure S15**. **Cumulative GC skew plot for the *Trichuris muris* mitogenome.**

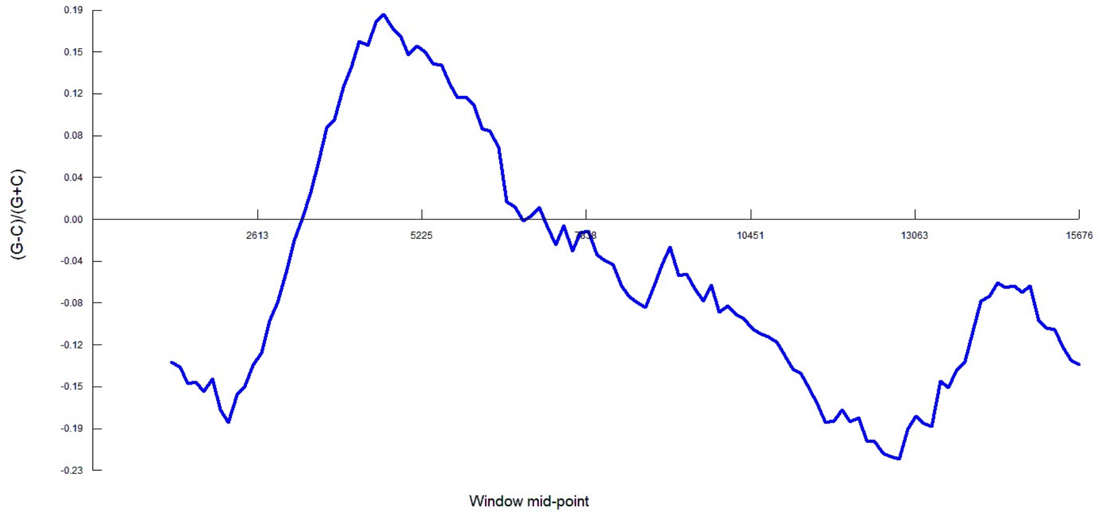


**Figure S16. Cumulative GC skew plot for the *Trichuris suis* mitogenome.**

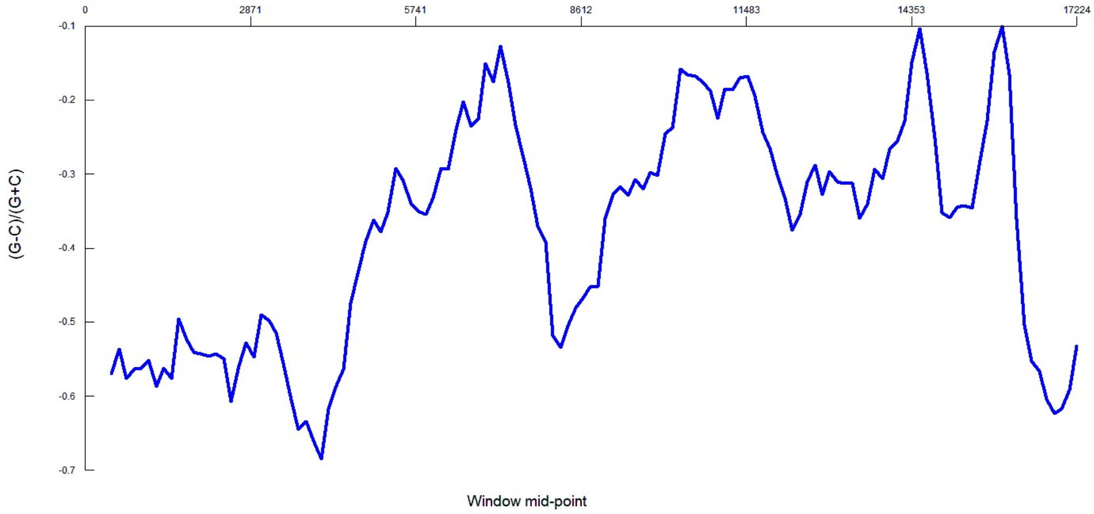


**Figure S17. Cumulative GC skew plot for the *Trichinella spiralis* mitogenome.**

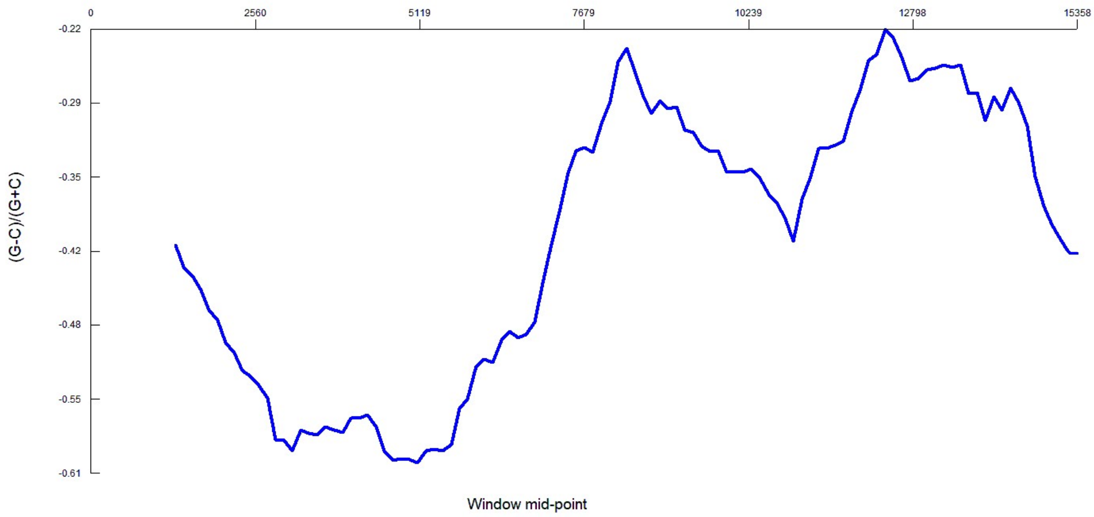


**Figure S18. Cumulative GC skew plot for the *Trichinella nativa* mitogenome.**

### SECTION I: HIGH-SCORING MOTIF OCCURENCES

- There were 4 motif occurences with a p-value less than 0.0001. The full set of motif occurences can be seen in the TSV (tab-delimited values) output file fimo.tsv, the GFF3 file fimo.gff which may be suitable for uploading to the UCSC Genome Table Browser (assuming the FASTA input sequences included genomic coordinates in UCSC or Galaxy format), or the XML file fimo.xml.
- The *p*-value of a motif occurrence is defined as the probability of a random sequence of the same length as the motif matching that position of the sequence with as good or better a score.
- The score for the match of a position in a sequence to a motif is computed by summing the appropriate entries from each column of the position-dependent scoring matrix that represents the motif.
- The q-value of a motif occurrence is defined as the false discovery rate if the occurrence is accepted as significant.
- The table is sorted by increasing *p*-value.

| Motif ID | Alt ID | Sequence Name | Strand | Start | End | p-value | q-value | Matched Sequence |
|---|---|---|---|---|---|---|---|---|
| 1 | GAGACCTGAGCCCAAGATA | misc_feature_NCR.fas_Xiphinema_americanum_NC_005928_1 | + | 66 | 84 | 3.01e-12 | 4.69e-07 | GAGACCTGAGCCCAAGATA |
| 1 | GAGACCTGAGCCCAAGATA | misc_feature_NCR.fas_Xiphinema_rivesi_NC_033869_1 | + | 66 | 84 | 1e-05 | 0.782 | GAAACCAGATCTCAAAATA |
| 1 | GAGACCTGAGCCCAAGATA | misc_feature_NCR_copy8.fas_Agamermis_sp__BH_2006_NC_008231_1 | - | 244 | 262 | 7.54e-05 | 1 | TATATTTGATCCCATGATA |
| 1 | GAGACCTGAGCCCAAGATA | misc_feature_NCR_copy9.fas_Romanomermis_iyengari_NC_008693_1 | - | 699 | 717 | 7.64e-05 | 1 | AAGACCTGAAATTAAAATA |

**Figure S19. FIMO search for the sequence motif 5′-GAGACCTGAGCCCAAGATA-3′.**

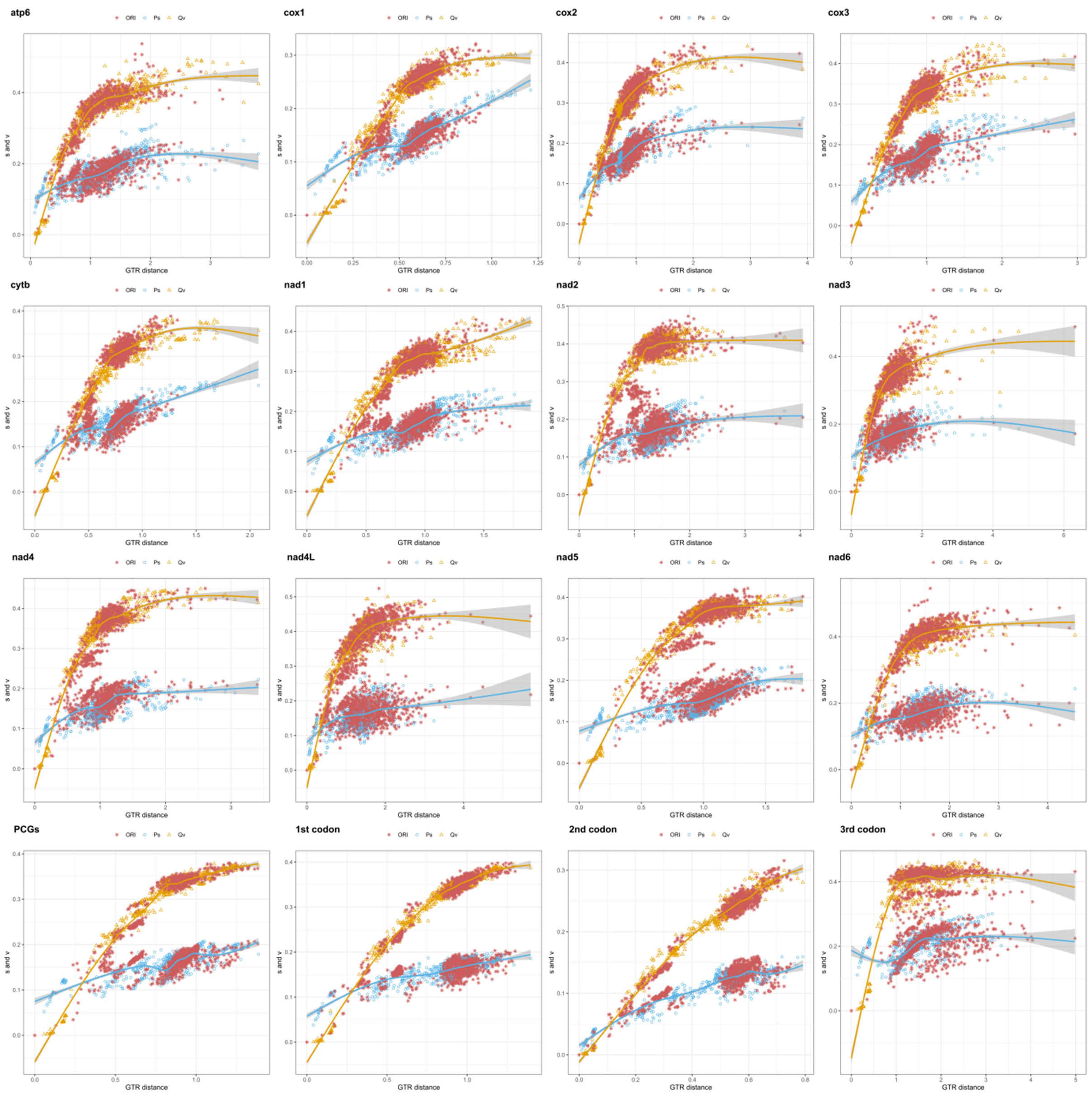


**Figure S20. Mutation saturation analyses for individual genes, concatenated genes (PCGs), and codon positions.**

Ps (yellow) refers to transitions and Qv to transversions (blue). ORI species are shown in red colour. The X-axis shows the GTR distance.

# Additional Methods

## Genome sequencing and assembly

After washing the sample in distilled water, DNA was isolated from the complete specimen using the AidLab DNA extraction kit (AidLab Biotechnologies, Beijing, China). PCR reaction mixture (50μL)

contained: 5U/μL TaKaRa LA Taq polymerase (TaKaRa, Japan), 10×LATaq Buffer II, 2.5μM dNTP mixture, 0.2-1.0μM each primer, and 60ng DNA template. PCR conditions were: denaturation 98°C/2min, 40 cycles of 98°C-10s, 50°C-15s, 68°C-1min/kb. When the product was not specific enough, PCR conditions were optimized by increasing the annealing temperature and decreasing the number of cycles. The PCR products were recovered by QIAquick Gel Extraction Kit (cat.No 28704), and then the purified PCR products were sent to Shanghai Sangong for Sanger sequencing (both uni- and bi-directional). Because the Sanger method is not suitable for sequencing amplicons longer than 900 bp, amplicons longer than 1 kbp were sequenced in several steps (Table S1). In each subsequent step, a new primer pair was designed on the basis of the obtained sequence. In cases when the F primer would have to align to a highly repetitive sequence (commonly AT runs), only new reverse primers were designed. After quality-proofing via visual inspection of electropherograms and identity confirmation using BLAST [9], the mitogenome was assembled manually using DNAstar v7.1 [10]. We made sure that overlaps were identical, the mitogenome circular, and that no *numt*s [11] were incorporated.

**Table S1. Additional primers used for the Sanger sequencing of amplified fragments larger than 1 kbp.** The total amplicon length in bp is given in brackets after the fragment name.

| Fragment | Step No. | Primer name | Sequence (5’-3’) |
|---|---|---|---|
| F2 (3395) | 1st | MX F1 | AACGTCTGTTCGACGTAAGA |
| | | MX R1 | CTACATCCATACCTACGGTG |
| | 2nd | MXF1-1 | GAGTTTATGAAGTTTTTGTC |
| | | MXR1-1 | CTGTAGATCGTCTAAAGAAAC |
| | 3rd | MXR1-2 | GAAAGGTATTATGGCAACCA |
| F4 (7150) | 1st | MX F2 | GATTGCCATGAATGATAGGA |
| | | MX R2 | CAAAATCTATATTCTACTTAAAC |
| | 2nd | MXF2-1 | GCAGATGTTATTCATAGTTG |
| | | MXR2-7 | CAATGATAATGTATGTAAGC |
| | 3rd | MXF1-1.5 | GCTGAATGTGGTCGAACTCC |
| | | MXR2-8 | GAGGCACCTGTAATAGGAAG |
| | 4th | MXR2-10 | CCTATGATATGATTTACTC |

| | | | |
|---|---|---|---|
| | 5th | MXR2-11 | GTTATTCTTAGGTTAATAC |
| | 6th | MXR2-12 | GCTGCTCCTACTCCTGTAAG |
| | 7th | MXR2-13 | GACATAATGTACACTTTTCC |
| | 8th | MXR2-14 | CATAGAAATTGAATATGAATCT |
| F5 (3469) | 1st | MX F3 | CAAAACCAATAATTCTGTGTG |
| | | MX R3 | TCTTACGTCGAACAGACGTT |
| | 2nd | MXF3-1 | GTTTTCTTGCATCTATGATG |
| | | MXR3-1 | CATAAAAGAGACATTTCTAATCC |
| | 3rd | MXR3-2 | GAGGGTGACGGGCGATATGTG |
| | 4th | MXR3-3 | GTATAACCGCGATAGCTGGCAC |

**Phylogenetic analyses**

For the mitochondrial phylogenomics analysis, duplicated PCGs comprised three *atp6* copies in *Hexamermis agrotis* (all three were identical), three *nad3* copies in *Romanomermis culicivorax* (all three were identical), and two *nad3* copies in *Romanomermis iyengari*, which was also the only duplication that exhibited difference between copies: position 41 (T vs. C). As this single mutation cannot affect our phylogenetic analysis in any meaningful way, we simply kept the copy 1 from all these duplicated genes.

For the *18S* analysis, all 245 available Capillariidae *18S* sequences were downloaded from GenBank, aligned using the FFT-NS-i algorithm in MAFFT, and then an ML tree was built using IQ-tree [12]. As these sequences varied in length and overlap, we pruned the alignment to 75 sequences that exhibited an almost full overlap with the newly sequenced 18S fragment with the help of MEGA X [13], and realigned the sequences. This produced an alignment with 2018 nucleotide sites, 1442 of which were invariant, 478 were parsimony informative, and there were 627 distinct site patterns. We selected the best model (TIM3e+R3) using ModelFinder [14], and reconducted the phylogenetic analysis with IQ-tree, using the selected model and 100,000 Ultrafast Bootstraps [15].

## References


1. Moravec F. Parasitic Nematodes of Freshwater Fishes of Europe. Springer Netherlands; 1994. https://www.springer.com/gp/book/9780792321729. Accessed 15 Jul 2021.

2. De Ley P, De Ley IT, Morris K, Abebe E, Mundo-Ocampo M, Yoder M, et al. An integrated approach to fast and informative morphological vouchering of nematodes for applications in molecular barcoding. Philosophical Transactions of the Royal Society B: Biological Sciences. 2005;360:1945–58. doi:10.1098/rstb.2005.1726.

3. Hebert PDN, Cywinska A, Ball SL, DeWaard JR. Biological identifications through DNA barcodes. Proceedings of the Royal Society of London Series B: Biological Sciences. 2003;270:313–21. doi:10.1098/rspb.2002.2218.

4. Derycke S, Vanaverbeke J, Rigaux A, Backeljau T, Moens T. Exploring the Use of Cytochrome Oxidase c Subunit 1 (COI) for DNA Barcoding of Free-Living Marine Nematodes. PLOS ONE. 2010;5:e13716. doi:10.1371/journal.pone.0013716.

5. Leis E, Easy R, Cone D. Report of the Potential Fish Pathogen Pseudocapillaria (Pseudocapillaria) tomentosa (Dujardin, 1843) (Nematoda) from Red Shiner (Cyprinella lutrensis) Shipped from Missouri to Wisconsin. copa. 2016;83:275–8. doi:10.1654/4821a.1.

6. Meldal BHM, Debenham NJ, De Ley P, De Ley IT, Vanfleteren JR, Vierstraete AR, et al. An improved molecular phylogeny of the Nematoda with special emphasis on marine taxa. Molecular Phylogenetics and Evolution. 2007;42:622–36.

7. Sakaguchi S, Yunus M, Sugi S, Sato H. Integrated taxonomic approaches to seven species of capillariid nematodes (Nematoda: Trichocephalida: Trichinelloidea) in poultry from Japan and Indonesia, with special reference to their 18S rDNA phylogenetic relationships. Parasitol Res. 2020;119:957–72. doi:10.1007/s00436-019-06544-y.

8. O'Leary NA, Wright MW, Brister JR, Ciufo S, Haddad D, McVeigh R, et al. Reference sequence (RefSeq) database at NCBI: Current status, taxonomic expansion, and functional annotation. Nucleic Acids Research. 2016;44:D733–45.

9. Altschul SF, Madden TL, Schäffer AA, Zhang J, Zhang Z, Miller W, et al. Gapped BLAST and PSI-BLAST: A new generation of protein database search programs. Nucleic Acids Research. 1997;25:3389–402.

10. Burland TG. DNASTAR's Lasergene sequence analysis software. In: Misener S, Krawetz SA, editors. Methods in Molecular Biology™. Totowa, NJ: Humana Press; 2000. p. 71–91. doi:10.1385/1-59259-192-2:71.

11. Hazkani-Covo E, Zeller RM, Martin W. Molecular poltergeists: Mitochondrial DNA copies (numts) in sequenced nuclear genomes. PLoS Genetics. 2010;6:e1000834.

12. Nguyen L-T, Schmidt HA, von Haeseler A, Minh BQ. IQ-TREE: A Fast and Effective Stochastic Algorithm for Estimating Maximum-Likelihood Phylogenies. Mol Biol Evol. 2015;32:268–74. doi:10.1093/molbev/msu300.

13. Kumar S, Stecher G, Li M, Knyaz C, Tamura K. MEGA X: Molecular Evolutionary Genetics Analysis across Computing Platforms. Mol Biol Evol. 2018;35:1547–9. doi:10.1093/molbev/msy096.

14. Kalyaanamoorthy S, Minh BQ, Wong TKF, Von Haeseler A, Jermiin LS. ModelFinder: Fast model selection for accurate phylogenetic estimates. Nature Methods. 2017;14:587–9.

15. Minh BQ, Nguyen MAT, von Haeseler A. Ultrafast Approximation for Phylogenetic Bootstrap. Molecular Biology and Evolution. 2013;30:1188–95. doi:10.1093/molbev/mst024.
