# Supplementary material for: Inverted base composition skews and discontinuous mitochondrial genome architecture evolution in the Enoplea (Nematoda)
Source: BMC Genomics. 2022 May 18;23:376. doi: 10.1186/s12864-022-08607-4 (PMC9115964; doi:10.1186/s12864-022-08607-4)
Supplement: Supplementary file 3 — Additional file 3. [file 12864_2022_8607_MOESM3_ESM.pdf]

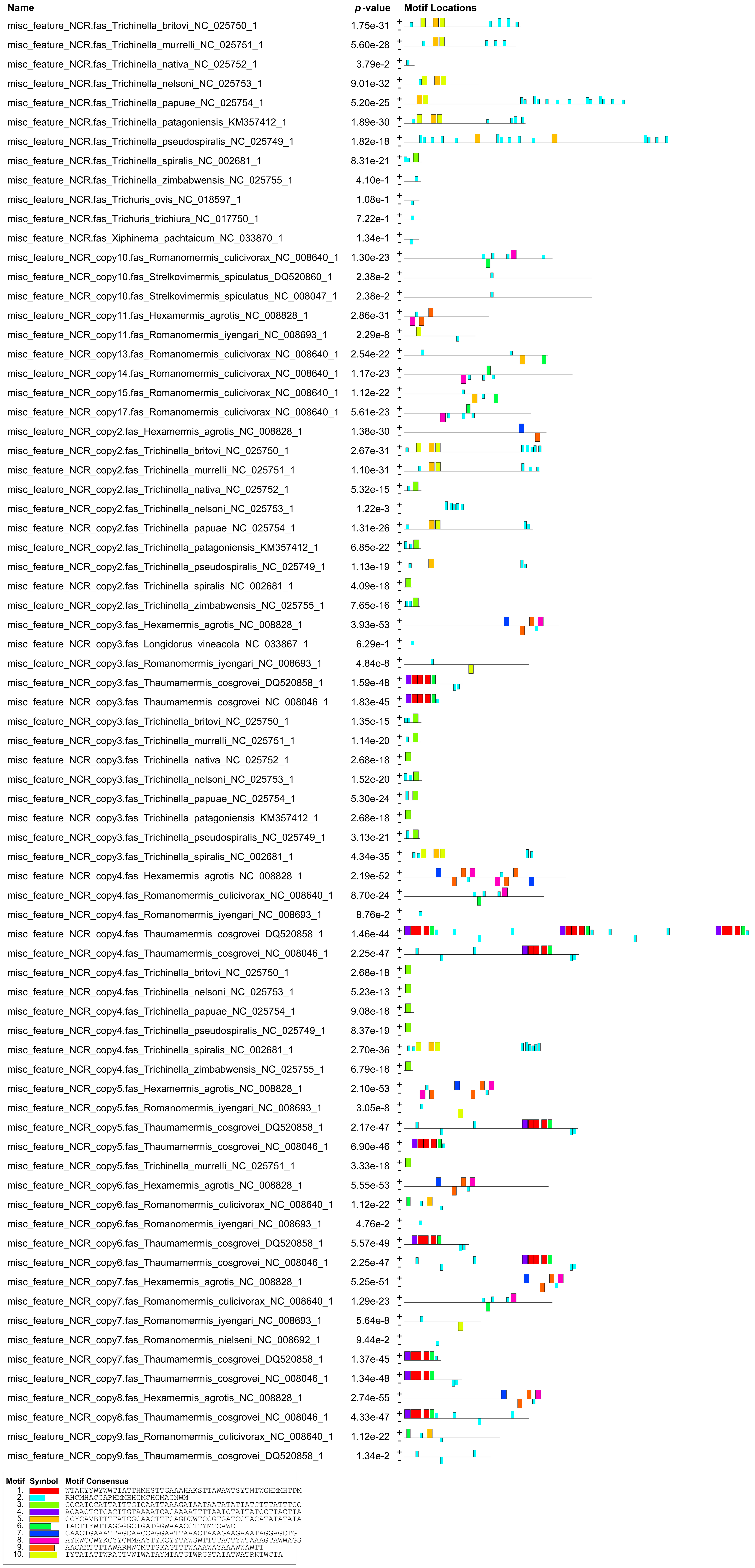

Additional file 3. Identification of conserved motifs in all NCRs ≥ 50 bp in the enoplean mitogenomes used for comparative analyses.
