# Supplementary material for: Inverted base composition skews and discontinuous mitochondrial genome architecture evolution in the Enoplea (Nematoda)
Source: BMC Genomics. 2022 May 18;23:376. doi: 10.1186/s12864-022-08607-4 (PMC9115964; doi:10.1186/s12864-022-08607-4)
Supplement: Supplementary file 4 — Additional file 4. [file 12864_2022_8607_MOESM4_ESM.pdf]

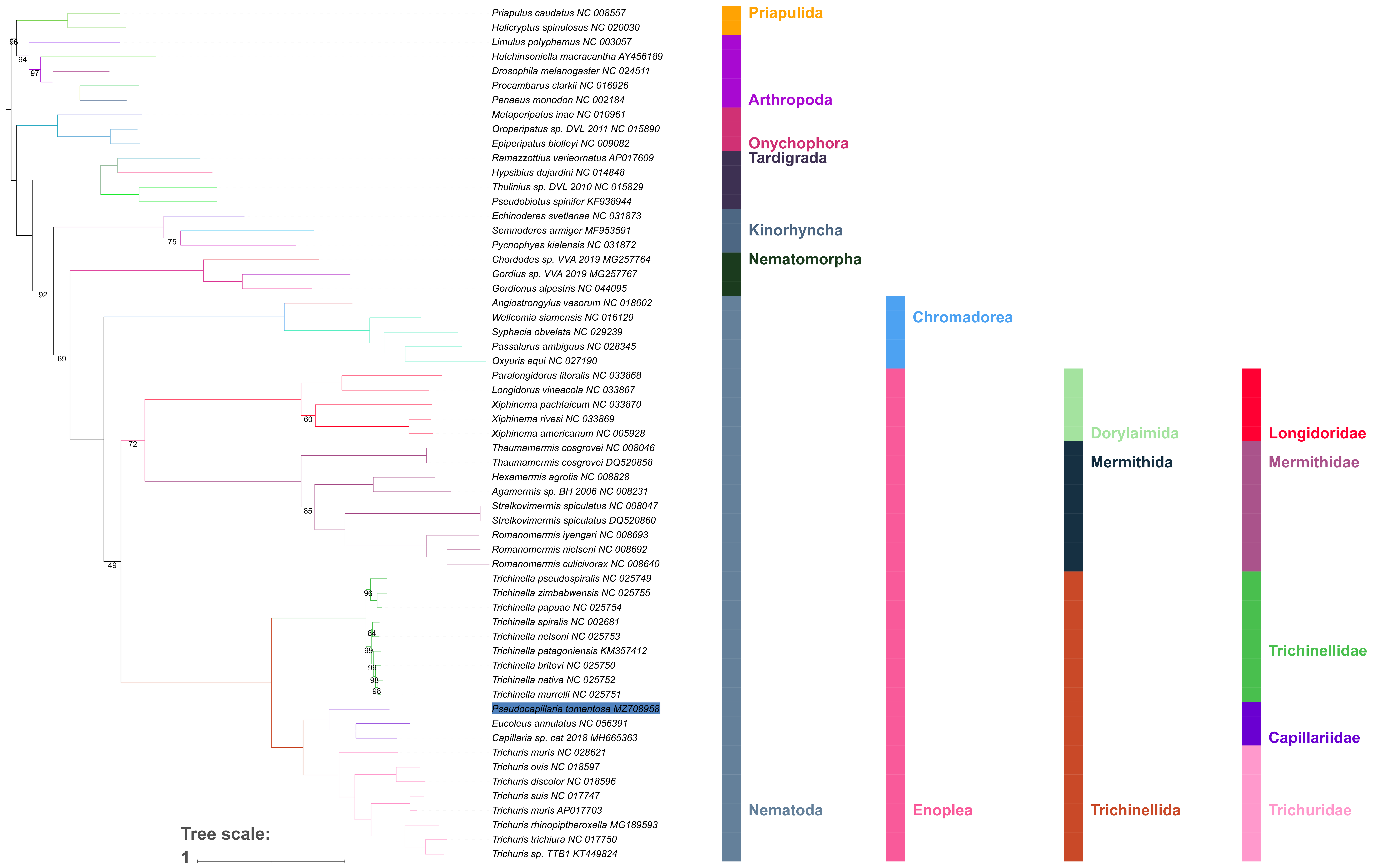


**Additional file 4. Mitochondrial phylogenomics of the Enoplea inferred using a dataset comprising nucleotide sequences of 36 mitochondrial genes.**

The phylogram was inferred using the Maximum Likelihood methodology implemented in IQ-TREE. Bootstrap support values are shown on the braches (only <100 values are shown). Species names are given with GenBank accession numbers. The phylum, class, order, and family-level taxonomic identities are shown to the right.
